# Liquid Ammonia: More than an Innocent Solvent for Zintl Anions

**DOI:** 10.1021/acs.inorgchem.4c01817

**Published:** 2024-08-09

**Authors:** Stefanie Gärtner, Michael Witzmann, Corinna Lorenz-Fuchs, Ruth M. Gschwind, Nikolaus Korber

**Affiliations:** † Institute of Inorganic Chemistry, University of Regensburg, 93053 Regensburg, Germany; ‡ Institute of Organic Chemistry, University of Regensburg, 93053 Regensburg, Germany

## Abstract

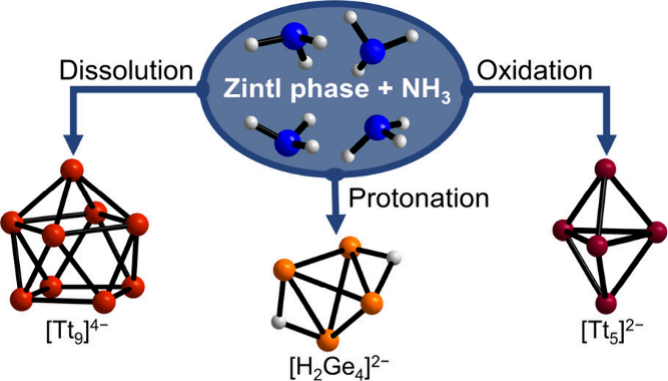

Liquid ammonia as the original solvent for Zintl anions has been replaced by easier to handle or more versatile solvents in most recent Zintl chemistry. However, methodological advances have made it possible to structurally investigate the anions in ammoniate crystals via crystallography or in the solutions themselves via nuclear magnetic resonance. While in some cases liquid ammonia acts as an innocent solvent, it also provides different possibilities of direct involvement in chemical reactions. In addition to simple dissolution without changes to the anions observed in the solid starting materials, protonation of the anion, incongruent dissolution involving redox processes, and further oxidation and reduction products have been observed. The use of the solvent liquid ammonia under ambient pressure is limited to low temperatures, which in turn allows the monitoring of kinetically stabilized species, some of which cannot be accessed at higher temperatures. In this work, the available literature reports are summarized or referenced, and compounds that have been characterized as new ammoniate crystals are presented and contextualized. Innocent dissolution is observed for clusters involved in K_2.9_Rb_5.1_[Si_4_][Si_9_]·15NH_3_, Cs_4_Sn_9_·12NH_3_, Cs_4_Pb_9_·5NH_3_, and [Rb@[18]crown-6]_2_[Rb@[2.2.2]crypt]Rb[Ge_9_]·4NH_3_. Formal protonation of [Ge_4_]^4–^ results in the crystallization of [Na@[2.2.2]crypt]_2_[H_2_Ge_4_]·3NH_3_. Tt_5_^2–^ (Tt = Sn or Pb) and HSi_9_^3–^ cannot be accessed in a binary solid state material but can be crystallized in co-crystals of PPh_3_ in [Rb@[2.2.2]crypt]_2_[Sn_5_][PPh_3_]_2_·NH_3_, [Rb@[2.2.2]crypt]_2_[Pb_5_][PPh_3_]_2_·NH_3_, and [K@[2.2.2]crypt]_3_[HSi_9_][PPh_3_]·5NH_3_.

## Abstract 2

Liquid ammonia is the original solvent in Zintl ion chemistry, but what happens upon dissolution of solid starting materials is not always straightforward. An overview of the different pathways observed is given, augmented and illustrated by crystal structures of several new ammoniate compounds of Zintl anions.

## Introduction

In 1891, A. Joannis reported on a green solution derived by the simultaneous dissolution of sodium metal and elemental lead in liquid ammonia.^1^ Although not being interpreted as such, this observation is the first documented evidence of a homoatomic polyanion of a main group metal produced in solution. Four decades later, E. Zintl produced and first structurally characterized the Zintl phase NaTl by reducing thallium(I) iodide in a solution of sodium in liquid ammonia.^2,3^ Again four decades later, Kummer and Diehl changed the solvent to ethylenediamine^4^ and Corbett introduced cryptand as a sequestering agent and reported the first clear crystal structure of a Zintl ion of a group 14 metal obtained by dissolution experiments.^4,5^ Although single crystals in liquid ammonia had been observed visually, these alterations were necessary for structural elucidation as X-ray structure analysis at that time was not possible for thermally labile crystals. Today, standard preparation techniques for labile crystals are applied widely for sensitive compounds and are used in all branches using this analytical method. In particular, the development of low-temperature devices and procedures allows for investigations of solvate crystals that decompose well below room temperature. For Zintl chemistry, this opened the door for investigation of very sensitive single crystals. In the following, we concentrate on the solvent with which everything had started, liquid ammonia. Upon closer examination of the reported species derived by (re)crystallization experiments in this solvent, it becomes evident that liquid ammonia might act as an innocent solvent but provides the capabilities of taking part in different reactions. In 1985, J. D. Corbett, to whom we owe major advances in the field of Zintl chemistry, stated, “The dependence of results on solvent has generally not been well explored.”^6^ In the past decades, a number of versatile compounds, which were obtained by various dissolution experiments and reactions, have been reported in review articles.^7−15^ The products are often unpredicted and surprising and emphasize the role of Zintl anions as a key to new homoatomic bonding motifs and interesting reactivities.^8,10,16−20^ The scope of this article is not to illustrate these well-reported issues. In contrast, we here go back to the roots and address a very general question. What reactivities of Zintl anions can be expected in solvent liquid ammonia? To obtain homoatomic p-block (semi)metal clusters in solution, there are different Zintl phases available. In general, the solubility of these materials in liquid ammonia is limited to compounds including alkali metals as less electronegative constituents. For group 14, A_4_Tt_4_ (A = Na–Cs, and Tt = Si–Pb),^21−29^ A_4_Tt_9_ (A = Na–Cs, and Tt = Ge–Pb),^30−33^ and A_12_Tt_17_ (A = Na–Cs, and Tt = Si–Pb)^32,34^ are generally used, while the solubility for silicides is commonly known to be best for A_12_Si_17_ materials.^35−38^ In accord with the Zintl–Klemm concept, these materials include tetrahedral [Tt_4_]^4–^ and/or monocapped square antiprismati- shaped [Tt_9_]^4–^ polyanions. A_12_Tt_17_ contains [Tt_9_]^4–^ and [Tt_4_]^4–^ clusters in a 1:2 ratio. For stannides, additionally A_52_Sn_82_ (A = K or Cs) is known, which also involves [Sn_4_]^4–^ and [Sn_9_]^4–^ clusters.^39^ It has to be noted that especially the materials of the heavier tetrelides suffer from low crystallinity due to disorder and the formation of plastic crystalline phases.^40,41^ Convenient representatives for dissolution experiments of group 15 Zintl phases are A_3_Pn_7_ (A = Na–Cs, and Pn = P–Sb),^42−47^ A_3_Pn_11_ (A = Na–Cs, and Pn = P–Sb),^48−50^ and A_4_Pn_6_ (A = Rb or Cs, and Pn = P or As).^51−53^ The A_3_Pn_7_ phases contain nortricyclane-shaped [Pn_7_]^3–^ clusters and the A_3_Pn_11_ phases tris-homocubane-shaped [Pn_11_]^3–^ (ufosan). In A_4_Pn_6_ planar, non-aromatic [Pn_6_]^4–^ anions^54^ represent the anionic entity.

The dissolution route uses the soluble Zintl phases mentioned above of the alkali metals, which are prepared by classical solid state methods and subsequently dissolved in liquid ammonia.^55^ The anions detected in the ammoniate crystals that precipitate allow conclusions to be drawn about the species present in the solution. It has to be pointed out that while detecting a Zintl anion in an ammoniate crystal is a very strong indication that it has been present in solution, this does not per se preclude the presence of additional different species or even rearrangements during the process of crystallization. Unfortunately, additional analytical methods are very difficult to apply due to the solvent being a condensed medium only below −33 °C at ambient pressure. Higher temperatures directly cause spontaneous vaporization. In this context, E. Zintl’s very accomplished experimental works have to be emphasized, as he performed potentiometric titrations in this solvent many decades ago.^3^ In recent years, attempts have succeeded in preparing nuclear magnetic resonance (NMR) probes for low-temperature measurements. Standard NMR probes with the coil for broadband detection located either as the inner or the outer coil provide large temperature ranges compatible with the requirements for liquid ammonia as the solvent and had been successfully applied in Zintl anion detection.^56,57^ The sensitivity of these probes is limited, but the large temperature range allows even for temperature-dependent studies down to 180 K. In contrast, the newer probes with cryogen-cooled coils provide excellent sensitivities for low-concentration species or slowly reacting ions.^58,59^

However, these cryoprobes have a reduced temperature range of ∼80 °C limiting low-temperature measurements to ≥240 K. These probes can be used only for elements for which NMR active isotopes are available. For homoatomic Zintl anion chemistry investigations in liquid ammonia solutions, ^31^P,^54,60^^29^Si,^36,56,57^ and ^119^Sn^36,61^ have been reported. Very recently, the possibility of detecting Raman spectra from ammoniate crystals in bomb tubes was shown,^62^ which might also be applicable for Zintl cluster ammoniates in the future. At the moment, information about ongoing processes during dissolution is based on solvate crystal structures and solution NMR spectroscopy where available. In the following, we provide an overview of the observed species during dissolution experiments of alkali metal tetrelides (Tt) and pnictogenides (Pn) in a liquid ammonia solution and new compounds K_2.9_Rb_5.1_[Si_4_][Si_9_]·15NH_3_ (**1**), Cs_4_Sn_9_·12NH_3_ (**2**), Cs_4_Pb_9_·5NH_3_, (**3**) [Rb@[18]crown-6]_2_[Rb@[2.2.2.]crypt]Rb[Ge_9_]·4NH_3_ (**4**), [Na@[2.2.2]crypt]_2_[H_2_Ge_4_]·3NH_3_ (**5**), [Rb@[2.2.2]crypt]_2_[Sn_5_][PPh_3_]_2_·NH_3_ (**6**), [Rb@[2.2.2]crypt]_2_[Pb_5_][PPh_3_]_2_·NH_3_ (**7**), and [K@[2.2.2]crypt]_3_[HSi_9_][PPh_3_]·5NH_3_ (**8**) are contextualized.

## Materials and Methods

### Synthesis

#### K_2.9_Rb_5.1_[Si_4_][Si_9_]·15NH_3_ (**1**)

First, 30 mg (0.025 mmol) of the solid state material with the nominal composition K_6_Rb_6_Si_17_ and 13 mg (0.013 mmol) of dibenzo-[18]crown-6 were dissolved in 5 mL of anhydrous liquid ammonia. The reddish orange solution was stored for nine months at 203 K, and orange needles of **1** could be isolated and characterized by single-crystal X-ray diffraction (SCXRD).

#### Cs_4_Sn_9_·12NH_3_ (**2**)

First, 200 mg (0.125 mmol) of the solid state material with the nominal composition Cs_4_Sn_9_ was dissolved in 5 mL of anhydrous liquid ammonia. The dark red solution was stored for five months at 203 K, and red crystals of **1** could be isolated and characterized by SCXRD. In addition, a solution of 200 mg of the nominal phase Cs_4_Sn_4_ in 5 mL of anhydrous liquid ammonia yielded crystals of **2** after storage at 203 K for five months.

#### Cs_4_Pb_9_·5NH_3_ (**3**)

First, 211 mg (0.848 mmol) of cesium, 389 mg (1.878 mmol) of lead, and 55 mg (0.207 mmol) of [18]crown-6 were dissolved in anhydrous liquid ammonia. After storage at 233 K for two months, black blocks of **3** could be isolated and characterized by SCXRD.

#### [Rb([18]crown-6)]_2_[Rb@[2.2.2]crypt]Rb[Ge_9_]·4NH_3_ (**4**)

First, 50 mg (0.022 mmol) of the solid state material with the nominal composition Rb_12_Ge_17_ and 12.4 mg (0.033 mmol) of [2.2.2]crypt were dissolved in anhydrous liquid ammonia, yielding a reddish brown solution. After storage at 203 K for four months, yellow needles of **4** could be isolated and characterized by SCXRD.

#### [Na@[2.2.2]crypt]_2_[H_2_Ge_4_]·3NH_3_ (**5**)

First, 50 mg (0.022 mmol) of the solid state material with the nominal composition Rb_12_Ge_17_, 22 mg (0.083 mmol) of [18]crown-6, and 19 mg (0.049 mmol) of [2.2.2]crypt were dissolved in anhydrous liquid ammonia, yielding a yellow solution. After storage at 203 K for four months, yellow crystals of **5** could be isolated and characterized by SCXRD.

#### [Rb@[2.2.2]crypt]_2_[Sn_5_][PPh_3_]_2_·NH_3_ (**6**)

First, 25 mg (0.030 mg) of the solid state material with the nominal composition Rb_4_Sn_4_, 12 mg (0.030 mmol) of [2.2.2]crypt, and 35 mg (0.030 mmol) of Pd(PPh_3_)_4_ were dissolved in anhydrous liquid ammonia. After storage at 233 K for six months, dark red blocks of **6** could be isolated and characterized by SCXRD.

#### [Rb@[2.2.2]crypt]_2_[Pb_5_][PPh_3_]_2_·NH_3_ (**7**)

First, 50 mg (0.040 mmol) of the solid state material with the nominal composition Rb_4_Pb_4_, 24.1 mg (0.060 mmol) of [2.2.2]crypt, and 21.1 mg (0.040 mmol) of Au(PPh_3_)Cl were dissolved in anhydrous liquid ammonia, yielding a dark green solution. After storage at 233 K for two months, dark violet plates of **7** could be isolated and characterized by SCXRD.

#### [K@[2.2.2]crypt]_3_[HSi_9_][PPh_3_]·5NH_3_ (**8**)

First, 30 mg (0.025 mmol) of the solid state material with the nominal composition K_6_Rb_6_Si_17_, 28 mg (0.073 mmol) of [2.2.2]crypt, 9 mg (0.037 mmol) of [18]crown-6, and 30 mg (0.025 mmol) of Pt(PPh_3_)_4_ were dissolved in anhydrous liquid ammonia, yielding an orange brown solution. After storage at 233 K for one year, yellow blocks of **8** could be isolated and characterized by SCXRD.

### Single-Crystal X-ray Diffraction

For the general procedure, see the Supporting Information for details regarding the individual compounds. All compounds are highly sensitive to moisture, air, and temperature. A small amount of crystals was transferred directly from the mother liquor from a cooled Schlenk vessel into liquid nitrogen stream-cooled perfluoroether oil. Suitable single crystals were isolated and subsequently transferred onto the goniometer using a MiTeGen loop cooled in liquid nitrogen during the transport. Data were collected at a temperature of 123 K with different diffractometers (see the Supporting Information for the respective setup used for the individual compounds). For data reduction, CrysAlisPro was used. Structure solution (ShelXT)^63^ and refinement (ShelXL)^64^ were performed in Olex2.^65^ The figures were created in Diamond 4^66^ using displacement ellipsoids at the 50% probability level.

## Results and Discussion

### Dissolution without Reaction

**Figure 1:**
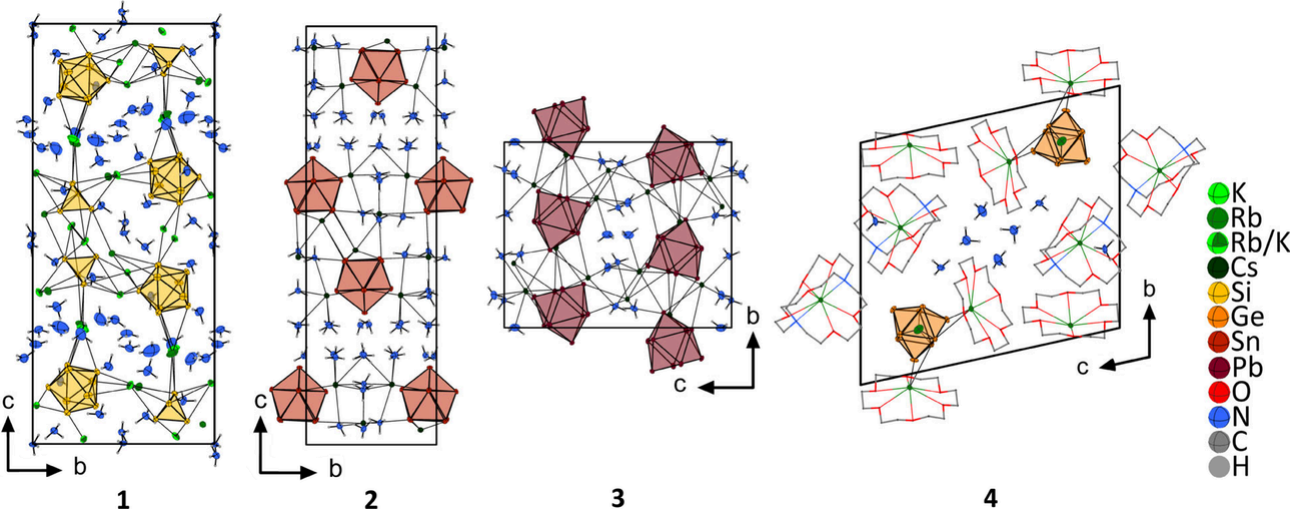
Unit cells of K_2.9_Rb_5.1_[Si_4_][Si_9_]·5NH_3_ (**1**), Cs_4_Sn_9_·12NH_3_ (**2**), Cs_4_Pb_9_·5NH_3_ (**3**), and [Rb@[18]crown-6]_2_[Rb@[2.2.2]crypt]Rb[Ge_9_]·5NH_3_ (**4**). For the sake of clarity, disorder and A–N (A = K or Rb) contacts in **1** have been omitted (for details, see Figure S2), whereas chelating agents in **4** are shown as wires and sticks.

The simplest process of generating Zintl anions in solution is represented by the dissolution of Zintl phases, including precast polyanions, where no subsequent reaction of the anions is monitored. The solubility of the Zintl salts can be influenced by applying different sequestering agents. For this reason, [18]crown-6 (1,4,7,10,13,16-hexaoxacyclooctadecane) or [2.2.2]cryptand (4,7,13,16,21,24-hexaoxa-1,10-diazabicyclo[8.8.8]hexacosane) is commonly used. The cryptates in return also facilitate crystallization, and these large molecular units significantly influence the observed three-dimensional structure in the solid state. Ammonia molecules of crystallization can act as rather innocent additives concerning solid state structures. Already in 1989, the room-temperature stable ammoniate Cs_3_(NH_3_)As_7_ was prepared and the comparison to Cs_3_As_7_ demonstrated the possibility of including ammonia of crystallization in binary Zintl phases.^67^ The obtained ammoniate is structurally related to the parental phase Cs_3_As_7_. A similar observation can be deduced for group 14 by comparing A_4_Tt_4_ (A = alkali metal, and Tt = Si–Pb) solid state phases and ammoniate structures.^68^ The first coordination sphere of the anion in the binaries and ammoniates is strongly related. Subsequently, several “pure” ammoniate crystal structures without further additives of [Tt_9_]^4–^, [Tt_4_]^4–^, [Pn_7_]^3–^, and [Pn_11_]^3–^ have been reported, all of which prove the innocent dissolution of the parental phases in liquid ammonia. In addition, it could be shown that [As_6_]^4–^ rings are retained in solution when dissolving binary phase Rb_4_As_6_.^60^ Heavier alkali metal cations K^+^–Cs^+^ tend to coordinate directly to the cluster units; therefore, usually three-dimensional cation–anion networks are observed. An example is given by K_2.9_Rb_5.1_[Si_4_][Si_9_]·15NH_3_ [**1** (Figure 1); for crystallographic information, see Table S1], which could be crystallized from solutions of silicide K_6_Rb_6_Si_17_ in liquid ammonia. The compound is structurally related to K_8_[Si_4_][Si_9_]·14.6NH_3_^69^ and demonstrates the possibility of substituting two potassium positions by rubidium (see Figure S1), while the remaining alkali cation sites are mixed occupied by potassium and rubidium.

In general, ammonia acting as a ligand toward the alkali cations is also capable of breaking the three-dimensional cation–anion network. This is usually the case when lithium or sodium is present, as homoleptic ammine complexes are formed. For example, in [Li(NH_3_)_4_]_3_As_7_·NH_3_ or [Li(NH_3_)_4_]_4_Sn_9_·NH_3_ and [Li(NH_3_)_4_]_4_Pb_9_·NH_3_ lithium–tetraammine complexes are present, which prevent direct cation–anion contacts.^70,71^ In contrast, Cs_4_Sn_9_·12NH_3_ (**2**) represents a new and first example in which the high ammonia content causes the formation of Cs^+^–[Sn_9_]^4–^ double layers built from cation–anion interactions, which are separated by ammonia molecules only [**2** (Figure 1); for crystallographic information, see Table S3]. To date, the formation of these types of layers in the respective crystal structures was observed only when cryptates or crown ethers were present during crystallization. Thus, Cs_4_Sn_9_·12NH_3_ shows the broad variety of possible arrangements in ammoniate crystal structures, even in the simplest case without additives. This is underlined by the crystal structure of Cs_4_Pb_9_·5NH_3_ (**3**), which indeed shows the expected three-dimensional cation–anion network [**3** (Figure 1); for crystallographic information, see Table S5]. As mentioned above, the addition of cryptating agents prevents the formation of a three-dimensional network. This is also well-reported for tetrelide cluster solvates of the solvent ethylenediamine.^8^ The same is true in liquid ammonia,^38,70,72^ and in the new example, [Rb@[18]crown-6]_2_[Rb@[2.2.2]crypt]Rb[Ge_9_]·4NH_3_ (**4**), the coordination motifs for Rb^+^ can be nicely demonstrated, as [Rb@[18]crown-6]^+^, [Rb@2.2.2crypt]^+^, and nonchelated Rb^+^ are present in the unit cell [**4** (Figure 1); for crystallographic information, see Table S7].

Altogether, recrystallization experiments suggest a certain stability of the precast [Tt_9_]^4–^, [Pn_7_]^3–^, and [Pn_11_]^3–^ anions in solution. This is also supported by ^119^Sn NMR of binary stannides in liquid ammonia. The [Sn_9_]^4–^ clusters are not affected by the solvent, as the well-known signal at approximately −1200 ppm is stable for a long time under these solutions.^61^ In contrast, the availability of [Sn_4_]^4–^ clusters in solutions of Rb_4_Sn_4_ can be monitored by its characteristic ^119^Sn signal at approximately −1800 ppm, which is observed only when cryptand is added or when the stannides are produced by an experimental procedure different from dissolution (direct reduction). This can be interpreted as a first hint that these clusters provide additional reactivities in a liquid ammonia solution. In general, ammoniate crystals of the ligand-free tetrahedral [Tt_4_]^4–^ clusters are rarely observed compared to the comparatively stable [Tt_9_]^4–^.^35,68,73,74^

### Reactions in and/or with Liquid Ammonia

The oxidation of Zintl phases is a chemically plausible and straightforward way to form bonds and the buildup of larger homoatomic entities, and different groups address this issue by various approaches under a range of experimental conditions.^75−80^ In particular, the oxidative coupling of homoatomic polyanions to form new elemental modifications is very promising.^81−83^ A prominent example is the oxidation of homoatomic clusters [Ge_9_]^4–^ in ionic liquids, as even a new elemental modification of germanium in terms of a guest-free germanium clathrate could be achieved.^84^ Furthermore, a variety of other novel mesoporous germanium materials can be obtained through the oxidation of such anionic clusters.^85−87^ Another example, which involves alkali metal Zintl phases, including cluster units, is the oxidation of K_4_Si_4_ under hydrogen pressure. There, reversible oxidation produces KSiH_3_, which is thus discussed as a promising material for hydrogen storage.^88^ The reactions with liquid ammonia at low temperatures allow the isolation of kinetically stabilized transitional compounds and therefore can yield precious information about ongoing processes during oxidation reactions. In general, one must distinguish among three reaction pathways. First, the clusters are in equilibrium with their oxidized counterparts and solvated electrons.^89^ Second, protonation of the clusters is possible due to the protic character of the solvent liquid ammonia (Protonation). Finally, incongruent dissolution can be observed for some Zintl phases, which yields new or formally oxidized homoatomic polyanions (Incongruent Dissolution). Different additives allow for the formation of interlinked clusters, the combination of both reaction pathways, or co-crystal formation (Further Examples of Oxidations and Reactions).

#### Protonation

**Figure 2:**
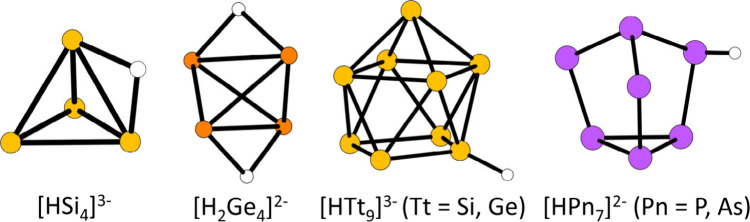
Overview of some of the known protonated tetrelides (Tt) and pnictogenides (Pn) from liquid ammonia.

Phosphorus hydrides including protonated Zintl clusters have thoroughly been studied by ^31^P NMR in different solvents.^90−93^ Additionally, HSn_9_^3–^^94^ and H_2_Si_9_^2–^^95^ in different solvents are reported in the literature. In liquid ammonia, protonation of homoatomic Zintl anions is known for silicon, germanium, phosphorus, and arsenic by single-crystal X-ray structures and/or solution NMR investigations. It has to be noted that protonation of the anion in this context is the formal reaction only. According to the electronegativities, the H atom in the protonated anion has to be considered as hydride, which is supported by the chemical shifts in related ^1^H NMR investigations.^56,57,91,94^ The Brønsted acid–base chemistry of pnictogenide clusters is discussed and summarized elsewhere;^13,14^ therefore, the focus here is set on the tetrelide clusters. Figure 2 shows examples for protonated tetrelide and pnictogenide clusters obtained from liquid ammonia solutions, and Table 1 gives examples for protonated tetrelides in and/or from liquid ammonia.

**Table 1:** Protonated Tetrelide Clusters in Liquid Ammonia

cluster	method or compound	ref
[HSi_4_]^3–^	^29^Si NMR	(57)
[H_2_Ge_4_]^2–^	[Na(crypt)]_2_[H_2_Ge_4_]·3NH_3_	this work
[HTt_9_]^3–^	(K(DB-[18]crown-6))(K@[2.2.2]crypt)_2_[HSi_9_]·8.5NH_3_	(56)
Tt = Si or Ge	[Rb@crypt]_2_[Rb([18]crown-6)][HGe_9_]·4NH_3_	(96)
	[K([18]crown-6)_3_(HSi_9_)]·2NH_3_·2THF^a^	(95)

a After dilution with tetrahydrofuran and exchange of NH_3_.

In general, the observation of protonated clusters in liquid ammonia seems to be restricted to the lighter homologues of a group, which is in accord with the rapidly decreasing stability of the element hydrides of the heavier congeners. While the ^31^P NMR investigations of M. Baudler mentioned above proved the formation of protonated phosphide clusters by the reduction of diphosphane in liquid ammonia,^90^ single crystals of the latter from liquid ammonia solutions were observed only when additives were present.^97−99^ In contrast, the protonation of silicides occurs spontaneously without the need for further additives. This is plausible given the higher charge of the tetrelides.

**Figure 3:**
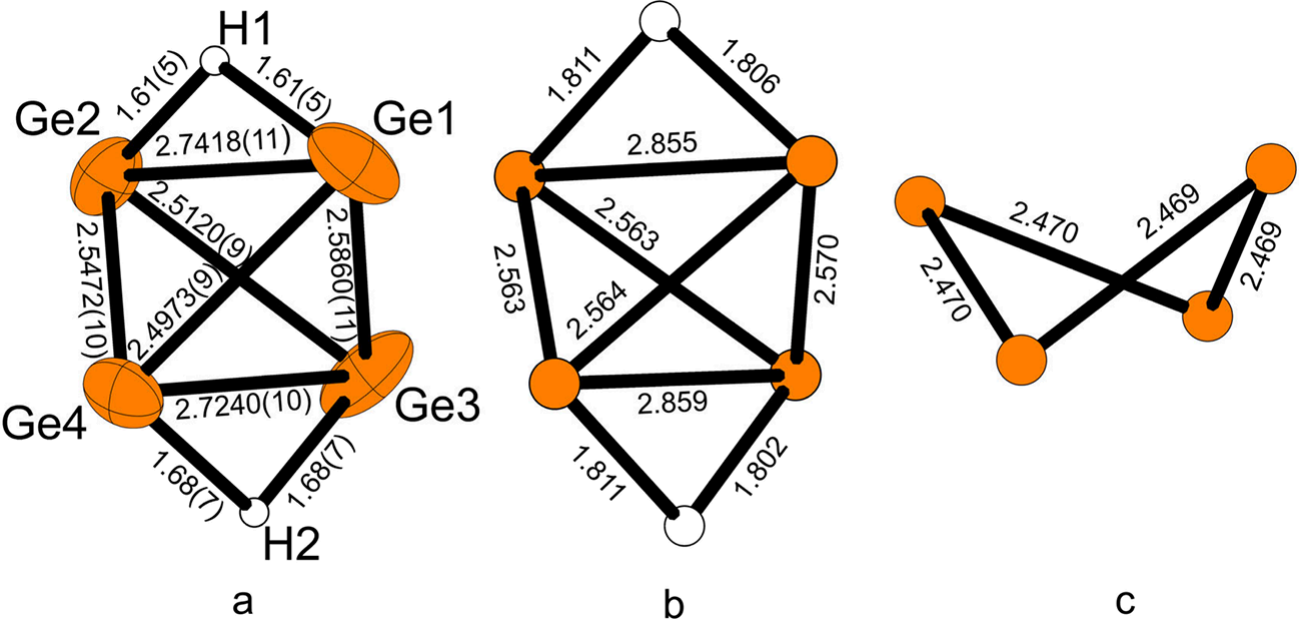
Atom arrangement and distances in [H_2_Ge_4_]^2–^ (a) from the crystal structure and (b) for an optimized geometry. (c) Optimized geometry of a hypothetical [Ge_4_]^2–^.

Attention needs to be drawn to protonated tetrahedral [Tt_4_]^4–^ species. While [HSi_4_]^3–^ was unambiguously detected in ^29^Si NMR experiments, a related crystal structure involving this anion is still missing. Interestingly, it was shown by the single-crystal structure of [K@[18]crown-6][Rb@[18]crown-6]_2_[HGe_4_ZnPh_2_]·8NH_3_^100^ that the related [HGe_4_]^3–^ can act as a ligand toward transition metals. To the best of our knowledge, similar observations of [HSi_4_]^3–^ are still missing. ^29^Si NMR solution studies and subsequent theoretical calculations suggest the protonation taking place not at one vertex of the tetrahedron but edge capping,^57^ just as in the heavier congener [HGe_4_]^3–^ in [HGe_4_ZnPh_2_]^2–^. This is also supported by calculations dealing with the solid state material K_2_BaSi_4_,^101^ which also suggest that the protonation of one vertex of [Si_4_]^4–^ is less favored. This is also observed for valence isoelectronic [HP_4_]^+^ cation with edge-capping hydrogen, according to the pseudoelement concept.^102,103^ Crystallization of ligand-free protonated tetrelides is very rarely observed. We recently succeeded in crystallizing [Na@[2.2.2]crypt]_2_[H_2_Ge_4_]·3NH_3_, which includes [H_2_Ge_4_]^2–^ anions. Unfortunately, the quality of the diffraction data of the SCXRD experiment is insufficient for determining the positions of the H atoms, which therefore have been modeled in the refinement using restraints. The positions of the Ge atoms yield distances that differ significantly from those of an ideal [Ge_4_]^4–^ tetrahedron (2.5–2.6 Å)^68^ as two edges are elongated (2.7418(11) and 2.7240(11) Å), which supports the structural interpretation. A first geometry optimization also strongly indicates a protonated species, as the optimized geometry for unprotonated [Ge_4_]^2–^ did not result in a tetrahedral anion but a strongly distorted four-atom ring. In contrast, the obtained Ge–Ge distances of the optimized geometry for [H_2_Ge_4_]^2–^ match the experimental values from the crystal structure very well (Figure 3; for computational details and crystallographic information see Table S9). Therefore, the overall charge of −2 together with the best structure solution from single-crystal data of the proposed model strongly supports the presence of [H_2_Ge_4_]^2–^ in liquid ammonia.

Upon closer examination of the unit cell parameters of [Na@[2.2.2]crypt]_2_[H_2_Ge_4_]·3NH_3_, a relationship with the (A@[2.2.2]crypt)_2_Tt_5_ compounds (Tt = Sn or Pb)^5,104,105^ that contain E_5_^2–^ anions becomes evident (Table 2).

**Table 2:** Unit Cell Parameters of (A@[2.2.2]crypt)_2_Tt_5_ and Related Compounds That Suggest Similar Three-Dimensional Arrangements

	X_2_Sn_5_^104^,^a^	X_2_Pb_5_^105^,^a^	X_2_[H_2_Ge_4_]·3NH_3_^a^	Z_2_Ge_5_·4NH_3_^106^,^b^
*a* (Å)	11.620(1)	11.615(3)	11.6200(6)	11.2887(9)
*b* (Å)	11.620(1)	11.615(3)	21.8720(7)	11.8949(9)
*c* (Å)	22.160(7)	22.120(12)	11.6979(6)	11.9433(9)
α (deg)	90	90	90	117.911(8)
β (deg)	90	90	119.469(6)	98.650(9)
γ (deg)	120	120	90	91.797(9)
crystal system, space group	trigonal, *P*3*c*1	trigonal, *P*3̅*c*1	monoclinic, *P*2_1_	triclinic, *P*1

a For the sake of clarity, the cryptand complexes have been replaced by X = (Na@[2.2.2]crypt).

b For the sake of clarity, the cryptand complexes have been replaced by Z = (K@[2.2.2]crypt).

While the ammonia-free compounds crystallize in high-symmetry trigonal space groups *P*3*c*1 and *P*3̅*c*1, respectively, ammonia of crystallization causes a lower observed symmetry. The cell parameters of [Na@[2.2.2]crypt]_2_[H_2_Ge_4_]·3NH_3_ still resemble a trigonal metric of the unit cell; in contrast, the symmetry in [K@[2.2.2]crypt]_2_Ge_5_·4NH_3_ is reduced even to triclinic *P*1 without any additional translational symmetry except identity.^106^

**Figure 4:**
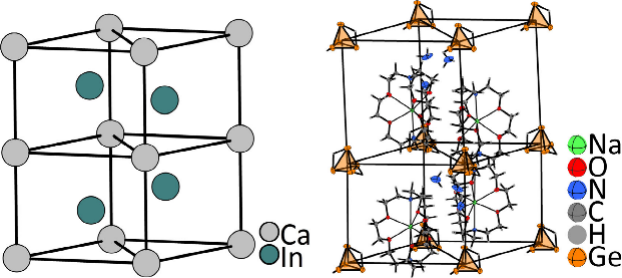
Structural similarity of CaIn_2_ and [Na@[2.2.2]crypt]_2_[H_2_Ge_4_]·3NH_3_.

In general, these compounds can be structurally related to binary CaIn_2_,^104^ and ammonia of crystallization causes a distortion in that arrangement in [A@[2.2.2]crypt]_2_Tt_5_·4NH_3_ (Tt = Si or Ge).^37^ A similar structural relationship can be visualized for [Na@[2.2.2]crypt]_2_[H_2_Ge_4_]·3NH_3_ (Figure 4). Of course, the atom positions differ significantly, which makes a comparison in terms of a formal isostructural derivation impossible.

The 2-fold negatively charged anion governs the 1:2 anion:cryptand ratio, which results in the best three-dimensional arrangement in the (distorted) CaIn_2_ packing. This is true for [Tt_5_]^2–^ (Tt = Si–Pb) as well as for [H_2_Ge_4_]^2–^ and demonstrates that for the evaluation of the observable anions in the solid state by recrystallization experiments the optimized packing in three dimensions should not be neglected.

#### Incongruent Dissolution

The central challenge for the clean generation of Zintl ions in solution from solid state starting materials is posed by the reactivity toward the solvent. In the case of liquid ammonia, the clusters mentioned in Protonation can be readily dissolved and (re)crystallized as ammoniates from the solutions, which indicates that they are at least somewhat stable toward this traditional Zintl ion solvent. In contrast, incongruent dissolution is also possible and common where the precast entity in the solid state transforms during dissolution. In these cases, mainly formally oxidized molecular units can be observed. With regard to group 14, incongruent dissolution is observed in NMR experiments for Rb_4_Sn_4_, which leads to [Sn_9_]^4–^ cage anions in the liquid ammonia solutions.^61^ Further evidence for this transformation from [Sn_4_]^4–^ to [Sn_9_]^4–^ is provided by the fact that Cs_4_Sn_9_·12NH_3_ (**2**) could also be crystallized from cryptand-free solutions of Cs_4_Sn_4_ (see Materials and Methods for **2**). In contrast, upon addition of [2.2.2]crypt, [Sn_4_]^4–^ anions can be detected as they accumulate and are stabilized in solution. The presence of [2.2.2]crypt in return enables the crystallization of trigonal bipyramidal-shaped [Sn_5_]^2–^ anions,^104,105,107^ for which no binary solid state material is known. The same is true for [Pb_5_]^2–^, which forms from solutions of Na_4_Pb_4_ and K_4_Pb_4_ in the presence of cryptand in liquid ammonia.^5,105^ The large cryptate complexes enable crystallization of [A@[2.2.2]crypt]_2_Tt_5_, which is hierarchically related to the CaIn_2_ structure type (see above). The lighter homologues yield [A@[2.2.2]crypt]_2_Tt_5_·4NH_3_ crystals from solutions of A_12_Tt_17_ materials (Tt = Si or Ge).^37,106,108^ In the crystal structures of the latter, additional ammonia of crystallization is needed for effective packing in lower-symmetry triclinic space groups. While [Tt_9_]^4–^ clusters are known to be more or less flexible on the NMR time scale,^56,94,95^ the ^29^Si NMR signal of [Si_5_]^2–^ recently proved the rigid character of the trigonal bipyramidal-shaped anion.^57^

Even approximately 50 years ago, the use of cryptand also provided the possibility of observing Sb_4_^2–^ and Bi_4_^2–^ anions crystallizing from not further characterized metallic alloys upon dissolution in ethylenediamine.^109,110^ The lighter homologues As_4_^2–^ and P_4_^2–^ can also be accessed by directly reducing red phosphorus or gray arsenic in liquid ammonia.^54,111−113^ Furthermore, the dissolution of A_4_Pn_6_ (A = Rb or Cs, and Pn = P or As) does not result in [P_6_]^4–^ but yields lone-pair aromatic [P_4_]^2–^ anions as a formal oxidation product. [As_6_]^4–^ is known to be more stable in solution as ammoniate crystals that contain this anion have been obtained (see Protonation). Additionally, dissolution of A_4_As_6_ (A = Rb or Cs) also provides formally oxidized As_7_^3–^^70^ and As_4_^2–^^54,111^ in solution.

Very recently, it was shown that group 13 Zintl phases Na_2_In and Na_7_KIn_4_ are oxidized upon dissolution in liquid ammonia to form binary NaIn. As Na_7_KIn_4_ includes tetrahedral Zintl anion [In_4_]^8–^, this might be interpreted as the first evidence for a solution behavior of trielides in anhydrous liquid ammonia similar to the reactivities of tetrelides or pnictogenides mentioned above.^114−116^

**Figure 5:**
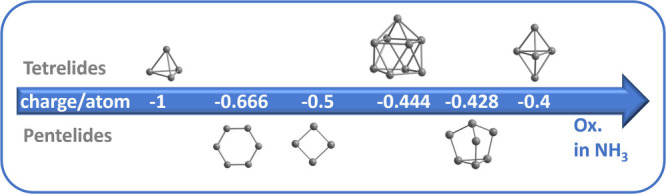
Clusters in tetrelide and pnictogenide Zintl phases in the dependency of charge per atom. Highly reduced clusters like [Tt_4_]^4–^ and [Pn_6_]^4–^ tend to dissolve incongruently, while the less reduced species like [Tt_9_]^4–^ or [Pn_7_]^3–^ are found to be stable in liquid ammonia solution.

Altogether, Zintl phases including more reduced cluster species tend to dissolve incongruently under the formation of formally oxidized cluster units in solution (Figure 5). Simultaneously, ammonia is reduced to form alkali metal amide and elemental hydrogen. In general, the side product alkali metal amide can be detected by NMR in solution as well as by PXRD of the residue after the evaporation of ammonia.

#### Further Examples of Oxidations and Reactions

**Figure 6:**
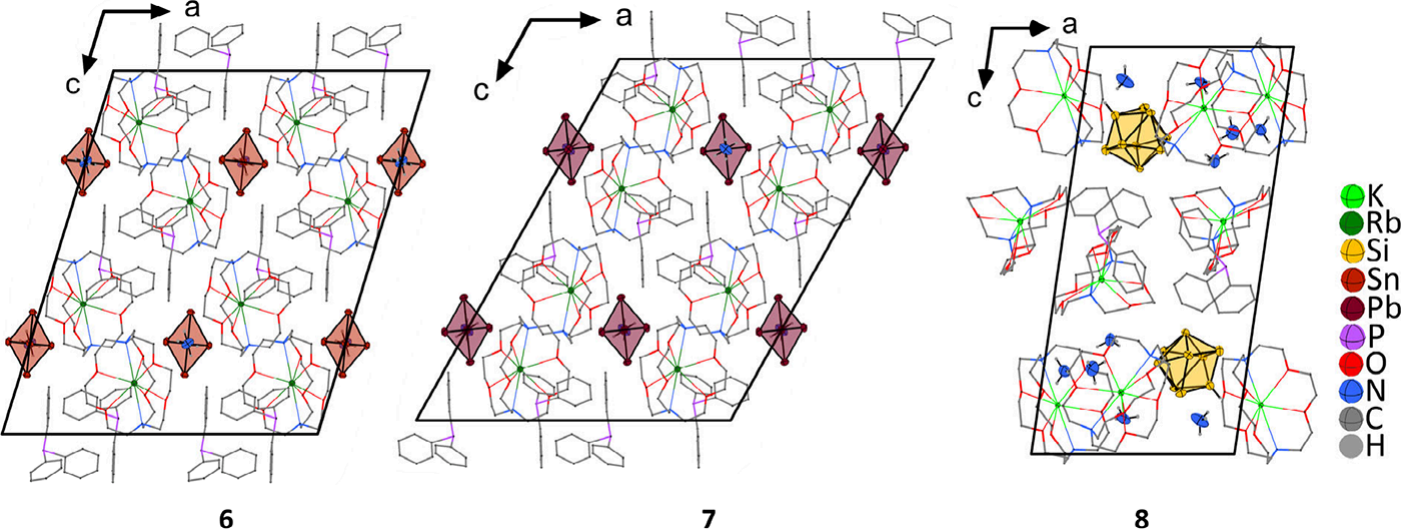
Projection of the unit cells of the compounds [Rb@[2.2.2]crypt]_2_[Sn_5_][PPh_3_]_2_·NH_3_ (**6**), [Rb@[2.2.2]crypt]_2_[Pb_5_][PPh_3_]_2_·NH_3_ (**7**), and [K@[2.2.2]crypt]_3_[HSi_9_][PPh_3_]·5NH_3_ (**8**) along the crystallographic *b*-axis. For the sake of clarity, [2.2.2]crypt and PPh_3_ are shown as wires and sticks.

The examples mentioned above are due to reactions with ammonia as no further additives except [18]crown-6 or cryptand were added. In the past, attention was drawn to reactions of the cluster units with different transition metal complexes. This gave rise to further observations, because co-crystallization is possible with, e.g., PPh_3_, as this well-known ligand molecule was present in solution due to the degradation of the complexes. In [Rb@[2.2.2]crypt]_2_[Sn_5_][PPh_3_]_2_·NH_3_ and [Rb@[2.2.2]crypt]_2_[Pb_5_][PPh_3_]_2_·NH_3_, PPh_3_ is present in addition to the cryptates in the unit cells and obviously further facilitates the crystallization of [Tt_5_]^2–^ upon incongruent dissolution of the Zintl phases Rb_4_Sn_4_ and Rb_4_Pb_4_, respectively. This is also true for [K@[2.2.2]crypt]_3_[HSi_9_][PPh_3_]·5NH_3_, where the protonated silicide is present in the unit cell (Figure 6; for crystallographic information about the compounds, see Tables S11, S13, and S15).

In contrast to [Na@[2.2.2]crypt]_2_[H_2_Ge_4_]·3NH_3_, in [Rb@[2.2.2]crypt]_2_[Sn_5_][PPh_3_]_2_·NH_3_ and [Rb@[2.2.2]crypt]_2_[Pb_5_][PPh_3_]_2_·NH_3_ the additive PPh_3_ causes an even more distorted structure that cannot be directly related to CaIn_2_ (for further information, see Figure S10). More systematic investigations of crystal packing in co-crystals with further additives like PPh_3_ could show if up to now hidden Zintl ions might be accessible in co-crystals.

**Figure 7:**
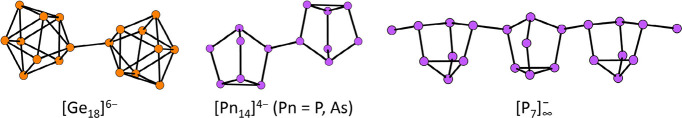
Overview of selected condensed tetrelide or pnictogenide clusters from liquid ammonia. A detailed overview for groups 14 and 15 is given in refs (14) and (18).

More severe conditions are necessary to observe the oxidative coupling of group 14 and group 15 clusters to form larger interlinked clusters, as these are obtained exclusively when additional oxidants are provided. The oxidative strength of the solvent liquid ammonia itself seems not to be sufficient for the formation of interlinked clusters like, e.g., [P_14_]^4–^^117^ or [Ge_18_]^6–^^118^ (Figure 7).

The observations of these units from liquid ammonia solution are very serendipitous and are not yet well understood. A very rare example is represented by CsP_7_, which is formed by the reaction of Cs_3_P_11_ and tellurium in liquid ammonia. CsP_7_ includes one-dimensional chains of linked P_7_ clusters, but no ammonia molecules of crystallization are present.^119^ This example emphasizes the large potential of liquid ammonia also for preparing binary compounds and alloys, which are difficult to access by classical solid state synthesis.

## Summary

Zintl anions in liquid ammonia make up a very fascinating and at the same time very challenging class of compounds, as their chemistry in solution is dependent on a filigree interplay of stabilization in solid state, solubility, and acidity. The documentation of results, obtained by crystallization and NMR in solution, allows one to gain further insights into ongoing processes. Slight changes in the experimental approach often result in a severe impact on the whole system. The results demonstrate that the common hypothesis of liquid ammonia as not only a historic but also a prototypic innocent solvent for Zintl anions needs to be revised. In other words, liquid ammonia must also be seen as a very potent reaction medium for yet not very well investigated ongoing oxidation processes of Zintl anions at low temperatures.

## Supporting Information Available

Computational details, additional synthetic details, crystallographic information and selected interatomic distances for all compounds, and structural comparison of **5–7** with CaIn_2_ (PDF)
