# Supplementary material for: Liquid Ammonia: More than an Innocent Solvent for Zintl Anions
Source: Inorg Chem. 2024 Aug 9;63(43):20240–9. doi: 10.1021/acs.inorgchem.4c01817 (PMC11523214; doi:10.1021/acs.inorgchem.4c01817)
Supplement: Supplementary file 1 — ic4c01817_si_001.pdf [file ic4c01817_si_001.pdf]

Supporting Information for:

Liquid Ammonia: More than an Innocent Solvent for Zintl Anions

Stefanie Gärtner[a], *Michael Witzmann* [a], *Corinna Lorenz-Fuchs* [a], *Ruth M. Gschwind* [b], *Nikolaus Korber*[a]*

a)Institute of Inorganic Chemistry, University of Regensburg, Regensburg, Germany; b)Institute of Organic Chemistry, University of Regensburg, Regensburg, Germany

*Corresponding authors email address: nikolaus.korber@ur.de

Computational details 1

Determination of yields 1

$K_{2.9}Rb_{5.1}[Si_4][Si_9] \cdot 15NH_3$ (1): 2

$Cs_4Sn_9 \cdot 12NH_3$ (2): 5

$Cs_4Pb_9 \cdot 5NH_3$ (3): 7

$[Rb@[18]crown\text{-}6]_2[Rb@[2.2.2]crypt]Rb[Ge_9] \cdot 4NH_3$ (4): 9

$[Na@[2.2.2]crypt]_2[H_2Ge_4] \cdot 3NH_3$ (5): 11

$[Rb@[2.2.2]crypt]_2[Sn_5][PPh_3]_2 \cdot NH_3$ (6): 13

$[Rb@[2.2.2]crypt]_2[Pb_5][PPh_3]_2 \cdot NH_3$ (7): 15

$[K@[2.2.2]crypt]_3[HSi_9][PPh_3] \cdot 5NH_3$ (8): 18

References 21

Computational details

All calculations were carried out by means of the program Orca 5.0[1,2], applying the B3LYP functional[3–6] and def2-TZVPP basis sets[7]. A CPCM model[8] with ammonia as the solvent was implemented.

Determination of yields and further characterization

The determination of yields was not possible for extremely air and temperature labile ammoniate crystals as they decompose spontaneously. Small crystalline amounts could only be handled in cooled perfluorinated oil before being transferred to the diffractometer for data collection and structure determination. Due to low solubilities, no further characterization by spectroscopic methods was possible.

$K_{2.9}Rb_{5.1}[Si_4][Si_9] \cdot 15NH_3$ (1):

Synthesis:

A solid state precursor with the nominal composition $K_6Rb_6Si_{17}$ (30 mg, 0.025 mmol), [2.2.2]crypt (0.013 mg, 0.014 mmol) and dibenzo-[18]crown-6 (0.013 mg, 0.013 mmol) were dissolved in 5 mL anhydrous liquid ammonia, yielding a reddish orange solution. After storage at 203 K for nine months, orange needles of (1) could be obtained.

Crystallographic information:

The crystal structure was recorded on an Agilent SuperNova with microfocus Cu-X-ray source and a large area Atlas CCD detector. The corresponding crystallographic data is listed in Table S1.

Table S1: Crystallographic data of $K_{2.9}Rb_{5.1}[Si_4][Si_9] \cdot 15NH_3$.

Chemical formula	$K_{2.9}Rb_{5.1}[Si_4][Si_9] \cdot 15NH_3$
CSD number	2330994
Formula weight	1166.95
Temperature/K	123(2)
Crystal system	orthorhombic
Space group	$P2_12_12_1$
a/Å	10.2984(2)
b/Å	13.7324(3)
c/Å	31.4084(7)
α/°	90
β/°	90
γ/°	90
Volume/Å^3	4441.83(16)
Z	4
ρ_{calc}g/cm^3	1.745
μ/mm^{-1}	12.934
F(000)	2299.0
Crystal size/mm^3	0.197 × 0.015 × 0.012
Radiation	Cu Kα (λ = 1.54184)
2Θ range/°	7.026 to 134.148
Index ranges	$-12 \leq h \leq 11$, $-16 \leq k \leq 16$, $-37 \leq l \leq 32$
Reflections collected	21053
Independent reflections	7812 [R_{int} = 0.0451, R_{sigma} = 0.0544]
Data/restraints/parameters	7812/54/418
Goodness-of-fit on F^2	1.021
Final R indexes [I>=2σ (I)]	R_1 = 0.0402, wR_2 = 0.0982
Final R indexes [all data]	R_1 = 0.0457, wR_2 = 0.1017
Largest diff. peak/hole / e Å^{-3}	1.35/-1.30
Flack parameter	0.008(12)

Supplementary figures and distances:

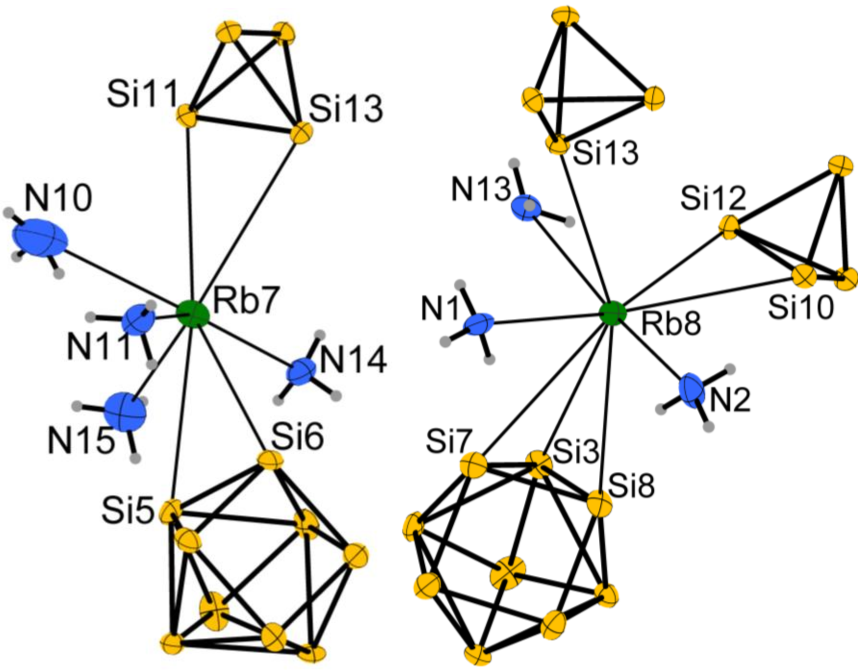

Figure S1: Coordination environment of the alkali metals Rb7 and Rb8 in $K_{2.9}Rb_{5.1}[Si_4][Si_9] \cdot 15NH_3$ with the corresponding distances in Table S2.

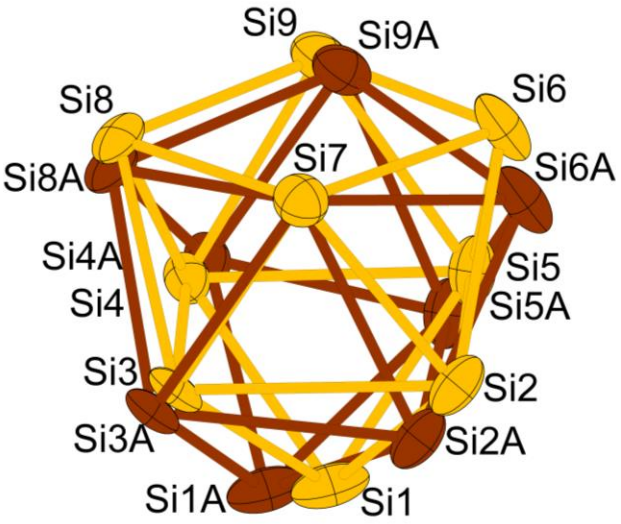

Figure S2: Main component (yellow) and disordered component (brown) of the $[Si_9]^{4-}$ anion in $K_{2.9}Rb_{5.1}[Si_4][Si_9] \cdot 15NH_3$ with occupation factors of 0.798(10) and 0.202(10) respectively, with the corresponding distances in Table S2.

Table S2: Selected interatomic distances in $K_{2.9}Rb_{5.1}[Si_4][Si_9] \cdot 15NH_3$.

Atom	Atom	Length/Å	Atom	Atom	Length/Å	Atom	Atom	Length/Å
Rb8	Si13	3.808(2)	Rb1	Si11	3.619(3)	Si3	Si2	2.590(11)
Rb8	Si12[1]	3.906(2)	Si13	Si12	2.423(3)	Si3	Si1	2.443(13)
Rb8	Si10[1]	3.767(2)	Si13	Si10	2.431(3)	Si4	Si5	2.633(9)
Rb8	Si7[2]	3.835(3)	Si13	Si11	2.403(3)	Si4	Si9	2.442(9)
Rb8	N1[3]	3.535(8)	Si13	Rb4	3.378(3)	Si4	Si1	2.435(9)
Rb8	N2[3]	3.106(8)	Si13	Rb3	3.406(3)	Si5	Si9	2.440(8)
Rb7	Si13	4.016(2)	Si12	Si10	2.396(3)	Si5	Si2	2.680(7)
Rb7	Si11	3.755(3)	Si12	Si11	2.423(3)	Si5	Si6	2.425(6)
Rb7	N14	3.179(8)	Si12	Rb3	3.500(3)	Si5	Si1	2.428(6)
Rb7	N15	3.059(11)	Si12	K5	3.650(2)	Si9	Si6	2.445(5)
Rb5	Si12	3.650(2)	Si12	Rb2	3.558(3)	Si2	Si6	2.457(6)
Rb5	Si7[4]	3.636(3)	Si10	Si11	2.439(3)	Si2	Si1	2.431(6)
Rb5	Si11	3.803(3)	Si10	Rb4	3.315(3)	N2	Rb3	2.987(9)
Rb5	N4	3.654(9)	Si10	Rb2	3.533(3)	N3	K5	3.050(8)
K6	Si7	3.613(3)	Si7	Si8	2.429(9)	N12	Rb4	3.376(8)
K6	Si7[4]	3.635(3)	Si7	Si3	2.361(8)	N7	Rb2	2.953(10)
K6	N12[5]	2.990(8)	Si7	Si2	2.420(5)	Si6A	Si2A	2.42(2)
K2	Si12	3.558(3)	Si7	Si6	2.417(4)	Si6A	Si5A	2.43(3)
K2	Si10	3.533(3)	Si7	Rb6	3.613(3)	Si6A	Si9A	2.41(3)
K2	Si11	3.476(3)	Si7	Si6A	2.497(19)	Si2A	Si1A	2.41(2)
K3	Si13	3.406(3)	Si7	Si2A	2.696(19)	Si2A	Si5A	2.65(4)
K3	Si13[1]	3.460(3)	Si7	Si8A	2.57(4)	Si2A	Si3A	2.45(5)
K3	Si12	3.500(3)	Si7	Si3A	2.60(4)	Si1A	Si5A	2.37(3)
K3	Si12[1]	3.531(3)	Si11	K1	3.461(8)	Si1A	Si4A	2.35(4)
K3	Si10[1]	3.679(3)	Si11	Rb4	3.768(3)	Si1A	Si3A	2.33(6)
K3	N1	3.033(8)	Si11	K5	3.803(3)	Si5A	Si9A	2.58(4)
K3	N2	2.987(9)	Si11	Rb2	3.476(3)	Si5A	Si4A	2.30(5)
K4	Si13	3.378(3)	Si8	Si3	2.440(13)	Si9A	Si8A	2.44(5)
K4	Si10	3.315(3)	Si8	Si4	2.430(9)	Si9A	Si4A	2.44(4)
K4	Si11	3.768(3)	Si8	Si9	2.435(11)	Si8A	Si4A	2.35(5)
K4	N12	3.376(8)	Si3	Si4	2.730(14)	Si8A	Si3A	2.41(6)
Rb1	Si10	3.765(3)						

[1]1/2+X,1/2-Y,1-Z; [2]+X,-1+Y,+Z; [3]-1/2+X,1/2-Y,1-Z; [4]1/2+X,3/2-Y,1-Z; [5]+X,1+Y,+Z

$Cs_4Sn_9 \cdot 12NH_3$ (2):

Synthesis:

A solid state material with the nominal composition Cs_4Sn_9 (200 mg, 0.125 mmol) was dissolved in anhydrous liquid ammonia. After storage at 203 K for five months, dark red blocks needles of (2) could be obtained. Crystals of the same compound were also observed for analogous preparation from a precursor with nominal composition Cs_4Sn_9 after five months.

Crystallographic information:

The crystal structure was recorded on an Agilent SuperNova with microfocus Mo-X-ray source and a large area Eos CCD detector. The corresponding crystallographic data is listed in Table S3.

Table S3: Crystallographic data of $Cs_4Sn_9 \cdot 12NH_3$.

Chemical formula	$Cs_4Sn_9 \cdot 12NH_3$
CSD number	2330993
Formula weight	1804.380
Temperature/K	123.00(10)
Crystal system	orthorhombic
Space group	$P2_12_12_1$
a/Å	10.0914(1)
b/Å	10.7705(1)
c/Å	34.2221(5)
α/°	90
β/°	90
γ/°	90
Volume/Å^3	3719.58(7)
Z	4
ρ_{calc}g/cm^3	3.222
μ/mm^{-1}	9.805
F(000)	3135.3
Crystal size/mm^3	0.23 × 0.16 × 0.09
Radiation	Mo Kα (λ = 0.71073)
2Θ range/°	6.02 to 63.02
Index ranges	$-14 \leq h \leq 14$, $-15 \leq k \leq 15$, $-49 \leq l \leq 50$
Reflections collected	66287
Independent reflections	11717 [R_{int} = 0.0765, R_{sigma} = 0.0428]
Data/restraints/parameters	11717/6/242
Goodness-of-fit on F^2	1.011
Final R indexes [I>=2σ (I)]	R_1 = 0.0305, wR_2 = 0.0667
Final R indexes [all data]	R_1 = 0.0331, wR_2 = 0.0680
Largest diff. peak/hole / e Å^{-3}	1.37/-1.26
Flack parameter	-0.033(19)

Interatomic distances

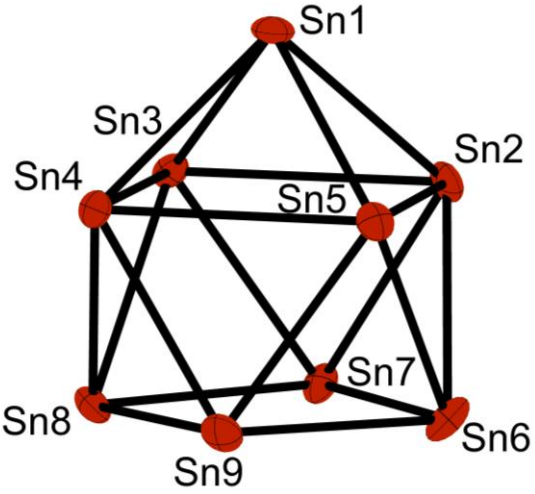

Figure S3: Anionic unit $[Sn_9]^{4-}$ in $Cs_4Sn_9 \cdot 12NH_3$, with the corresponding distances in Table S4.

Table S4: Selected interatomic distances in $Cs_4Sn_9 \cdot 12NH_3$.

Atom	Atom	Length/Å	Atom	Atom	Length/Å	Atom	Atom	Length/Å
Cs1	Sn4[2]	4.4098(5)	Cs4	N12	3.538(8)	Sn4	Sn1	2.9462(6)
Cs1	Sn6	3.7659(6)	Sn3	Sn4	3.2567(6)	Sn6	Sn7	2.9660(6)
Cs1	Sn6[3]	4.0257(5)	Sn3	Sn7	2.9648(5)	Sn6	Sn5	2.9481(6)
Cs1	Sn7	4.1201(5)	Sn3	Sn2	3.1678(5)	Sn6	Sn2	2.9586(5)
Cs1	Sn2	4.2352(5)	Sn3	Sn8	2.9507(6)	Sn6	Sn9	2.9233(5)
Cs1	Sn8[2]	4.2772(6)	Sn3	Sn1	2.9543(5)	Sn6	Cs2[1]	3.9552(5)
Cs1	Sn9[3]	4.0659(6)	Cs3	Sn4[6]	4.2301(6)	Sn7	Sn2	2.9626(5)
Cs1	Sn9[2]	3.8108(6)	Cs3	Sn2[3]	4.1092(5)	Sn7	Sn8	2.9649(6)
Cs1	N2[1]	3.469(5)	Cs3	N8	3.317(5)	Sn7	Cs2[8]	4.1221(6)
Cs1	N1	3.259(6)	Cs3	N2	3.327(5)	Sn5	Sn2	3.2088(6)
Cs4	Sn4[4]	4.2117(6)	Cs3	N5	3.385(6)	Sn5	Sn9	2.9608(6)
Cs4	Sn2[5]	4.1111(6)	Cs3	N4	3.346(5)	Sn5	Sn1	2.9481(5)
Cs4	N8	3.358(5)	Cs3	N6	3.289(6)	Sn2	Sn1	2.9435(5)
Cs4	N5	3.338(6)	Cs3	N9	3.259(6)	Sn8	Sn9	2.9656(6)
Cs4	N4	3.350(5)	Cs3	N12A[7]	3.66(2)	Sn8	Cs2[9]	3.9365(6)
Cs4	N7	3.308(6)	Sn4	Sn5	3.1854(6)	Cs2	N2	3.399(5)
Cs4	N10	3.309(6)	Sn4	Sn8	2.9443(6)	Cs2	N4	3.218(5)
Cs4	N3	3.377(6)	Sn4	Sn9	2.9566(6)			

[1] 1/2+X,1/2-Y,1-Z; [2] -1+X,+Y,+Z; [3] -1/2+X,1/2-Y,1-Z; [4] -3/2+X,3/2-Y,1-Z; [5] -1/2+X,3/2-Y,1-Z; [6] -3/2+X,1/2-Y,1-Z; [7] +X,-1+Y,+Z; [8] 1/2+X,3/2-Y,1-Z; [9] 1+X,+Y,+Z

$Cs_4Pb_9 \cdot 5NH_3$ (3):

Synthesis:

Cesium (211 mg, 0.848 mmol), lead (389 mg, 1.878 mmol) and [18]crown-6 (55 mg, 0.207 mmol) were dissolved in anhydrous liquid ammonia. After storage at 233 K for two months, black blocks of (3) could be obtained.

Crystallographic information:

The crystal structure was recorded on an Agilent Gemini ultra with microfocus Mo-X-ray source and a large area AtlasS2 CCD detector. The corresponding crystallographic data is listed in Table S5.

Table S5: Crystallographic data of $Cs_4Pb_9 \cdot 5NH_3$.

Chemical formula	$Cs_4Pb_9 \cdot 5NH_3$
CSD number	2330994
Formula weight	2481.52
Temperature/K	123(2)
Crystal system	monoclinic
Space group	$P2_1/c$
a/Å	9.8784(7)
b/Å	15.4803(10)
c/Å	19.1757(11)
α/°	90
β/°	91.630(6)
γ/°	90
Volume/Å^3	2931.2(3)
Z	4
ρ_{calc}g/cm^3	5.623
μ/mm^{-1}	56.377
F(000)	4032.0
Crystal size/mm^3	0.278 × 0.178 × 0.177
Radiation	Mo Kα (λ = 0.71073)
2Θ range/°	6.406 to 52.744
Index ranges	$-12 \leq h \leq 12$, $-19 \leq k \leq 19$, $-23 \leq l \leq 23$
Reflections collected	29884
Independent reflections	5981 [R_{int} = 0.0921, R_{sigma} = 0.0762]
Data/restraints/parameters	5981/6/163
Goodness-of-fit on F^2	0.988
Final R indexes [I>=2σ (I)]	R_1 = 0.0397, wR_2 = 0.0641
Final R indexes [all data]	R_1 = 0.0654, wR_2 = 0.0705
Largest diff. peak/hole / e Å^{-3}	2.05/-1.93

Interatomic distances

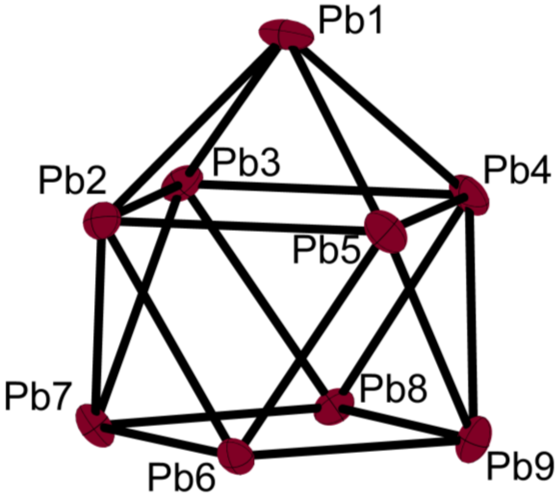

Figure S4: Anionic unit $[Pb_9]^{4-}$ in $Cs_4Pb_9 \cdot 5NH_3$, with the corresponding distances in Table S6.

Table S6: Selected interatomic distances in $Cs_4Pb_9 \cdot 5NH_3$.

Atom	Atom	Length/Å	Atom	Atom	Length/Å	Atom	Atom	Length/Å
Pb8	Pb3	3.0698(8)	Pb7	Cs2	4.0822(12)	Pb4	Cs3[1]	4.7234(12)
Pb8	Pb7	3.1228(9)	Pb7	Cs1	4.3195(12)	Pb4	Cs1[4]	4.6442(12)
Pb8	Pb9	3.0910(8)	Pb2	Pb5	3.3037(9)	Cs3	Cs3[5]	5.1016(19)
Pb8	Pb4	3.1060(9)	Pb2	Pb1	3.0994(9)	Cs3	Cs4[6]	4.7087(14)
Pb8	Cs3[1]	4.1256(11)	Pb2	Cs4[2]	4.0253(12)	Cs3	Cs2	4.3279(14)
Pb8	Cs1	3.8403(12)	Pb2	Cs1[2]	4.0206(12)	Cs3	Cs1	4.3318(14)
Pb3	Pb7	3.1179(9)	Pb9	Pb5	3.1562(9)	Cs3	N3[6]	3.323(14)
Pb3	Pb2	3.3839(8)	Pb9	Pb4	3.0682(9)	Cs3	N2	3.296(13)
Pb3	Pb1	3.0667(9)	Pb9	Cs4[3]	3.9980(12)	Cs3	N5	3.450(13)
Pb3	Pb4	3.3436(9)	Pb9	Cs2[3]	3.8437(11)	Cs4	Cs2[1]	4.4116(14)
Pb3	Cs3[1]	4.0243(11)	Pb9	Cs1	4.0502(12)	Cs4	Cs2	5.4566(14)
Pb3	Cs3[2]	4.0884(11)	Pb5	Pb1	3.0675(9)	Cs4	Cs1[2]	5.5670(14)
Pb3	Cs1[2]	3.8322(12)	Pb5	Pb4	3.3831(9)	Cs4	N3	3.428(14)
Pb6	Pb7	3.0705(8)	Pb5	Cs4[2]	4.5107(12)	Cs4	N5[1]	3.342(13)
Pb6	Pb2	3.0961(8)	Pb5	Cs2[3]	4.2945(12)	Cs4	N4[7]	3.199(15)
Pb6	Pb9	3.0979(9)	Pb5	Cs1[4]	3.7848(12)	Cs2	Cs1[2]	5.3866(15)
Pb6	Pb5	3.0587(9)	Pb1	Pb4	3.0849(9)	Cs2	N3[6]	3.320(14)
Pb6	Cs2[3]	4.2092(12)	Pb1	Cs3[2]	4.2266(12)	Cs2	N5	3.539(14)
Pb6	Cs1	4.5042(12)	Pb1	Cs4[2]	3.9601(12)	Cs2	N4[8]	3.301(15)
Pb7	Pb2	3.1096(9)	Pb1	Cs2[2]	4.0629(12)	Cs2	N1[9]	3.199(18)

[1]+X,1/2-Y,-1/2+Z; [2]-X,1/2+Y,1/2-Z; [3]1+X,+Y,+Z; [4]1-X,1/2+Y,1/2-Z; [5]-X,-Y,1-Z; [6]+X,1/2-Y,1/2+Z; [7]-1+X,+Y,+Z; [8]-1+X,1/2-Y,1/2+Z; [9]-X,1-Y,1-Z

$[Rb@[18]crown\text{-}6]_2[Rb@[2.2.2]crypt]Rb[Ge_9] \cdot 4NH_3$ (4):

Synthesis:

A solid state material with the nominal composition $Rb_{12}Ge_{17}$ (50 mg, 0.022 mmol), [18]crown-6 (14.5 mg, 0.055 mmol) and [2.2.2]crypt (12.4 mg, 0.033 mmol) were dissolved in anhydrous liquid ammonia, yielding a reddish brown solution. After storage at 203 K for four months, yellow needles of (4) could be obtained.

Crystallographic information:

The crystal structure was recorded on an Agilent SuperNova with microfocus Mo-X-ray source and an Eos CCD detector. The corresponding crystallographic data is listed in Table S7.

Table S7: Crystallographic data of $[Rb@[18]crown\text{-}6]_2[Rb@[2.2.2]crypt]Rb[Ge_9] \cdot 4NH_3$.

Chemical formula	$[Rb(18\text{-crown-}6)]_2[Rb([2.2.2]crypt)]Rb[Ge_9] \cdot 4NH_3$
CSD number	2330989
Formula weight	1968.43
Temperature/K	123.00(10)
Crystal system	triclinic
Space group	*P*-1
a/Å	9.9817(4)
b/Å	18.1164(7)
c/Å	20.4460(8)
α/°	101.820(3)
β/°	101.261(3)
γ/°	91.067(3)
Volume/Å^3	3542.6(2)
Z	2
ρ_{calc}g/cm^3	1.845
μ/mm^{-1}	6.553
F(000)	1940.0
Crystal size/mm^3	0.213 × 0.09 × 0.043
Radiation	Mo Kα (λ = 0.71073)
2Θ range/°	6.136 to 56.564
Index ranges	$-12 \leq h \leq 13$, $-24 \leq k \leq 24$, $-27 \leq l \leq 27$
Reflections collected	44222
Independent reflections	17524 [R_{int} = 0.0615, R_{sigma} = 0.0884]
Data/restraints/parameters	17524/0/712
Goodness-of-fit on F^2	1.007
Final R indexes [I>=2σ (I)]	R_1 = 0.0453, wR_2 = 0.0628
Final R indexes [all data]	R_1 = 0.0857, wR_2 = 0.0721
Largest diff. peak/hole / e Å^{-3}	0.81/-0.73

Interatomic distances

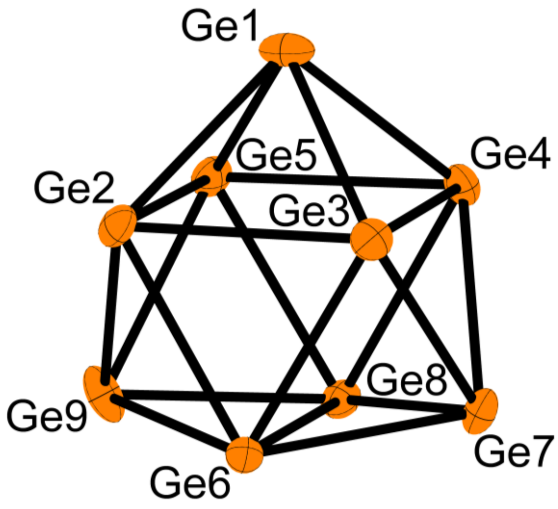

Figure S5: Anionic unit $[Ge_9]^{4-}$ in $[Rb@[18]crown\text{-}6]_2[Rb@[2.2.2]crypt]Rb[Ge_9] \cdot 4NH_3$, with the corresponding distances in Table S8.

Table S8: Selected interatomic distances in $[Rb@[18]crown\text{-}6]_2[Rb@[2.2.2]crypt]Rb[Ge_9] \cdot 4NH_3$.

Atom	Atom	Length/Å	Atom	Atom	Length/Å	Atom	Atom	Length/Å
Rb4	O16	2.874(3)	Rb1	Ge7	3.4393(6)	Ge6	Rb3[1]	3.6574(7)
Rb4	O13	2.869(3)	Rb1	O4	2.890(3)	Ge4	Ge5	2.6719(6)
Rb4	O17	2.896(3)	Rb1	O2	2.914(3)	Ge4	Ge7	2.5850(7)
Rb4	O18	2.869(3)	Rb1	O6	2.893(3)	Ge4	Ge1	2.5690(7)
Rb4	O14	2.884(3)	Rb1	O1	3.052(3)	Ge4	Rb3	3.5655(7)
Rb4	O15	2.866(3)	Rb1	O3	3.158(3)	Ge5	Ge1	2.5947(7)
Rb4	N5	3.048(3)	Rb1	O5	3.046(3)	Ge5	Ge9	2.6168(7)
Rb4	N6	2.970(3)	Ge8	Ge4	2.6639(7)	Ge5	Rb3	4.0369(7)
Rb2	Ge9	3.3949(7)	Ge8	Ge5	2.6031(7)	Ge2	Ge3	2.6917(7)
Rb2	O9	2.919(3)	Ge8	Ge7	2.5658(7)	Ge2	Ge1	2.5740(7)
Rb2	O12	3.119(3)	Ge8	Ge9	2.5968(6)	Ge2	Ge9	2.5810(7)
Rb2	O11	2.915(3)	Ge8	Rb3	3.7123(7)	Ge2	Rb3[1]	3.6237(7)
Rb2	O7	2.876(3)	Ge6	Ge2	2.6485(7)	Ge3	Ge7	2.6143(7)
Rb2	O8	3.030(3)	Ge6	Ge3	2.6230(7)	Ge3	Ge1	2.6004(7)
Rb2	O10	3.064(3)	Ge6	Ge7	2.5886(6)	Ge3	Rb3[1]	4.0944(7)
Rb1	Ge6	3.6567(6)	Ge6	Ge9	2.5669(7)	Rb3	O6[2]	3.621(3)
Rb1	Ge3	3.8713(7)						

[1]1+X,+Y,+Z; [2]-1+X,+Y,+Z

$[Na@[2.2.2]crypt]_2[H_2Ge_4] \cdot 3NH_3$ (5):

Synthesis:

A solid phase precursor with the nominal composition $Rb_{12}Ge_{17}$ (50 mg, 0.022 mmol), [18]crown-6 (22 mg, 0.083 mmol) and [2.2.2]crypt (19 mg, 0.049 mmol) were dissolved in anhydrous liquid ammonia, yielding a yellow solution. After storage at 203 K for four months, yellow crystals of (5) could be obtained.

Crystallographic information:

The crystal structure was recorded on an Agilent SuperNova with microfocus Mo-X-ray source and an Eos CCD detector. The corresponding crystallographic data is listed in Table S9. The fact that the crystallographic a and b axes are of similar length and that the β angle is close to 120° suggests a solution in a hexagonal cell. This was attempted and gave nonsensical structure solutions and refinement parameters. A monoclinic solution was therefore chosen.

Table S9: Crystallographic data of $[Na@[2.2.2]crypt]_2[H_2Ge_4] \cdot 3NH_3$.

Chemical formula	$[Na@[2.2.2]crypt]_2[H_2Ge_4] \cdot 3NH_3$
CSD number	1575849
Formula weight	1142.43
Temperature/K	123.15
Crystal system	monoclinic
Space group	$P2_1$
a/Å	11.6200(6)
b/Å	21.8720(7)
c/Å	11.6979(6)
α/°	90
β/°	119.469(6)
γ/°	90
Volume/Å^3	2588.4(2)
Z	2
ρ_{calc}g/cm^3	1.466
μ/mm^{-1}	2.376
F(000)	1188.0
Crystal size/mm^3	0.39 × 0.16 × 0.07
Radiation	MoKα (λ = 0.71073)
2Θ range/°	6.9 to 52.744
Index ranges	$-14 \leq h \leq 14$, $-27 \leq k \leq 27$, $-14 \leq l \leq 14$
Reflections collected	37846
Independent reflections	10553 [R_{int} = 0.0560, R_{sigma} = 0.0614]
Data/restraints/parameters	10553/7/788
Goodness-of-fit on F^2	1.034
Final R indexes [I>=2σ (I)]	R_1 = 0.0419, wR_2 = 0.0729
Final R indexes [all data]	R_1 = 0.0606, wR_2 = 0.0789
Largest diff. peak/hole / e Å^{-3}	0.61/-0.78
Flack parameter	-0.006(5)

Interatomic distances

Table S10: Selected interatomic distances in [Na@[2.2.2]crypt]$_2$[H_2Ge_4] · 3NH_3.

Atom	Atom	Length/Å	Atom	Atom	Length/Å	Atom	Atom	Length/Å
Ge1	H1	1.61(5)	Na2	O9	2.600(4)	Na1	O4	2.766(4)
Ge2	H1	1.61(5)	Na2	O8	2.793(5)	Na1	N4	2.725(5)
Ge3	H2	1.68(7)	Na2	N7	2.713(5)	Ge1	Ge4	2.4973(9)
Ge4	H2	1.68(7)	Na1	N5	3.253(7)	Ge1	Ge2	2.7418(11)
Na2	N6	3.210(5)	Na1	O5	2.697(4)	Ge1	Ge3	2.5861(11)
Na2	O11	2.481(4)	Na1	O2	2.466(4)	Ge4	Ge2	2.5472(10)
Na2	O7	2.505(4)	Na1	O1	2.586(4)	Ge4	Ge3	2.7240(10)
Na2	O10	2.501(4)	Na1	O3	2.456(4)	Ge2	Ge3	2.5124(11)
Na2	O12	2.780(5)	Na1	O6	2.468(4)			

Unit cell and structural comparison to $CaIn_2$

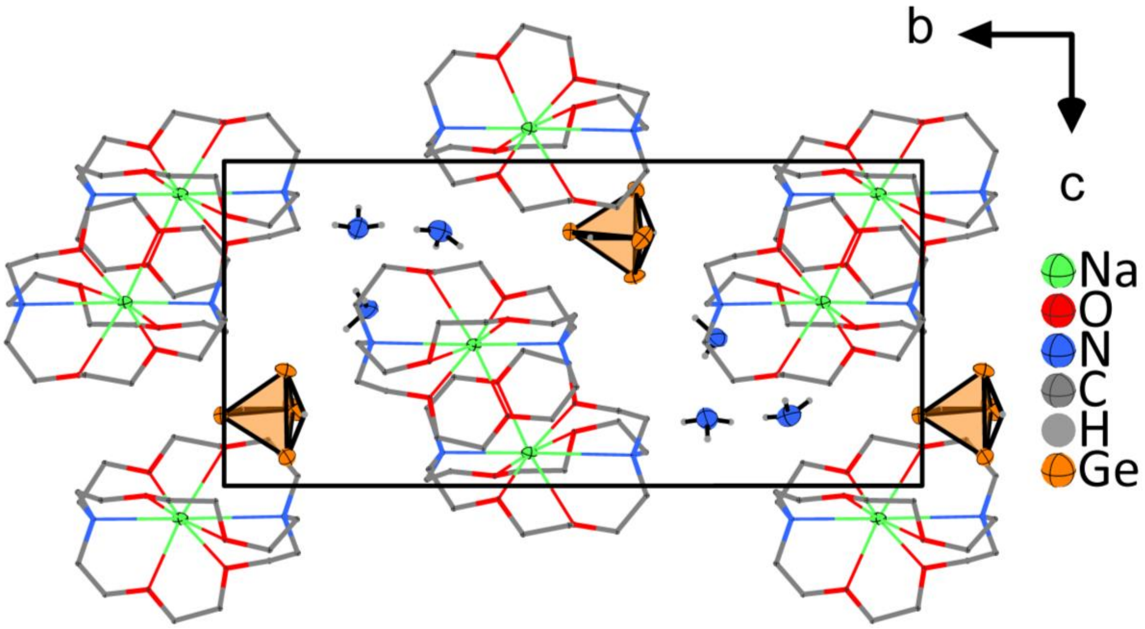

Figure S6: Unit cell of [Na@[2.2.2]crypt]$_2$[H_2Ge_4] · 3NH_3 along the crystallographic a-axis. For clarity, [2.2.2]crypt is shown as wires and sticks.

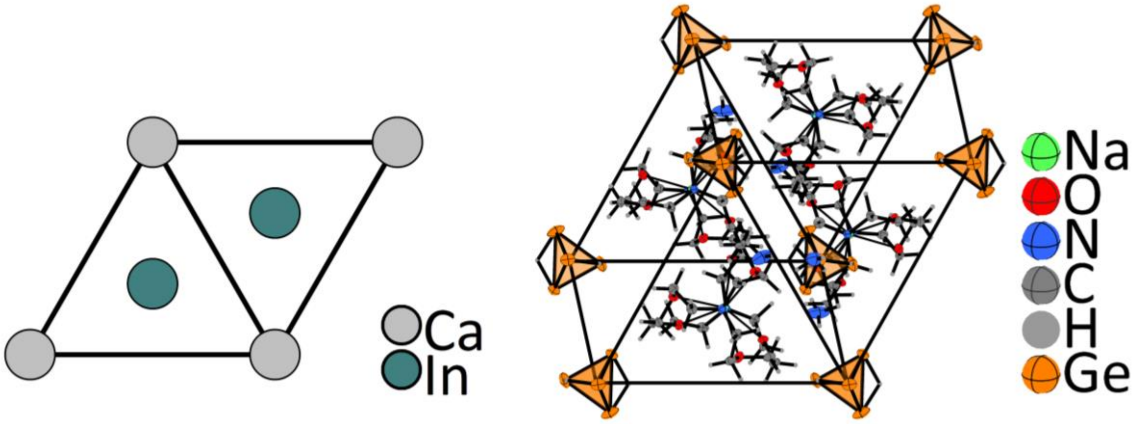

Figure S7: Structural comparison between $CaIn_2$ and $[Na@[2.2.2]crypt]_2[H_2Ge_4] \cdot 3NH_3$, visualizing the distorted hexagonal packing in $[Na@[2.2.2]crypt]_2[H_2Ge_4] \cdot 3NH_3$. The shown section of the crystal structure is not comparable to the unit cell.

$[Rb@[2.2.2]crypt]_2[Sn_5][PPh_3]_2 \cdot NH_3$ (6):

Synthesis:

A solid state material with the nominal composition Rb_4Sn_4 (25 mg, 0.030 mmol), [2.2.2]crypt (12 mg, 0.030 mmol) and $Pd(PPh_3)_4$ (35 mg, 0.030 mmol) were dissolved in anhydrous liquid ammonia. After storage at 233 K for six months, dark red blocks of (6) could be obtained.

Crystallographic information:

The crystal structure was recorded on an Agilent SuperNova with microfocus Mo-X-ray source and an Eos CCD detector. The corresponding crystallographic data is listed in Table S11.

Table S11: Crystallographic data of $[Rb@[2.2.2]crypt]_2[Sn_5][PPh_3]_2 \cdot NH_3$.

Chemical formula	$[Rb@[2.2.2]crypt]_2[Sn_5][PPh_3]_2 \cdot NH_3$
CSD number	2330991
Formula weight	2058.93
Temperature/K	123.15
Crystal system	monoclinic
Space group	*C*2/*c*
a/Å	22.6705(4)
b/Å	14.1020(2)
c/Å	27.1966(6)
α/°	90
β/°	107.138(2)
γ/°	90
Volume/Å^3	8308.7(3)
Z	4
ρ_{calc}g/cm^3	1.646
μ/mm^{-1}	2.742
F(000)	4088.0
Crystal size/mm^3	0.1265 × 0.0967 × 0.0778
Radiation	Mo Kα (λ = 0.71073)
2Θ range/°	6.118 to 51.362
Index ranges	$-27 \leq h \leq 26$, $-17 \leq k \leq 17$, $-33 \leq l \leq 23$
Reflections collected	15742
Independent reflections	7872 [R_{int} = 0.0208, R_{sigma} = 0.0349]
Data/restraints/parameters	7872/0/647
Goodness-of-fit on F^2	1.027
Final R indexes [I>=2σ (I)]	R_1 = 0.0255, wR_2 = 0.0421
Final R indexes [all data]	R_1 = 0.0363, wR_2 = 0.0453
Largest diff. peak/hole / e Å^{-3}	0.38/-0.31

Interatomic distances

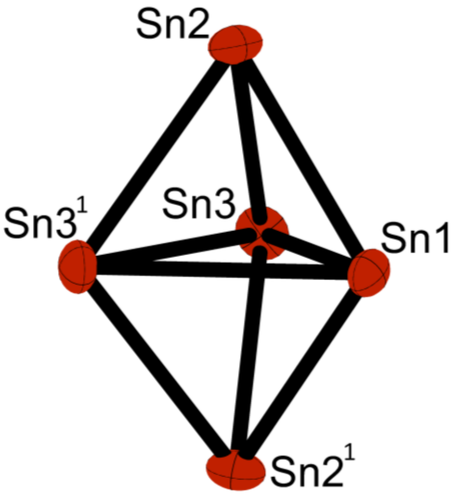

Figure S8: Anionic unit $[Sn_5]^{2-}$ in $[Rb@[2.2.2]crypt]_2[Sn_5][PPh_3]_2 \cdot NH_3$, with the corresponding distances in Table S12. Symmetry code #1: −X,+Y,1/2−Z.

Table S12: Selected interatomic distances in $[Rb@[2.2.2]crypt]_2[Sn_5][PPh_3]_2 \cdot NH_3$.

Atom	Atom	Length/Å	Atom	Atom	Length/Å	Atom	Atom	Length/Å
Sn1	Sn3	3.0791(3)	Sn3	Sn2	2.8713(3)	Rb1	O2	2.9011(18)
Sn1	$Sn3^1$	3.0791(3)	Sn3	$Sn2^1$	2.8704(3)	Rb1	O6	2.9124(17)
Sn1	$Sn2^1$	2.8757(3)	Rb1	O4	2.8564(17)	Rb1	O1	2.8672(18)
Sn1	Sn2	2.8757(3)	Rb1	O5	2.8499(17)	Rb1	N2	3.005(2)
Sn3	$Sn3^1$	3.0882(4)	Rb1	O3	2.8934(17)	Rb1	N1	3.006(2)

^{1}-X,+Y,1/2-Z

$[Rb@[2.2.2]crypt]_2[Pb_5][PPh_3]_2 \cdot NH_3$ (7):

Synthesis:

A solid state material with the nominal composition Rb_4Pb_4 (50 mg, 0.040 mmol), [2.2.2]crypt (24.1 mg, 0.060 mmol) and $Au(PPh_3)Cl$ (21.1 mg, 0.040 mmol) were dissolved in anhydrous liquid ammonia, yielding a dark green solution. After storage at 233 K for two months, dark violet plates of (7) could be obtained.

Crystallographic information:

The crystal structure was recorded on an Agilent SuperNova E with microfocus Mo-X-ray source and an Eos CCD detector. The corresponding crystallographic data is listed in Table S13.

Table S13: Crystallographic data of $[Rb@[2.2.2]crypt]_2[Pb_5][PPh_3]_2 \cdot NH_3$.

Chemical formula	$[Rb@[2.2.2]crypt]_2[Pb_5][PPh_3]_2 \cdot NH_3$
CSD number	2330990
Formula weight	2501.43
Temperature/K	123(2)
Crystal system	monoclinic
Space group	*C*2/*c*
a/Å	22.6544(8)
b/Å	14.1802(3)
c/Å	29.7630(10)
α/°	90
β/°	119.435(4)
γ/°	90
Volume/Å^3	8327.0(5)
Z	4
ρ_{calc}g/cm^3	1.995
μ/mm^{-1}	11.332
F(000)	4728.0
Crystal size/mm^3	0.286 × 0.179 × 0.089
Radiation	MoKα (λ = 0.71073)
2Θ range/°	6.658 to 61.366
Index ranges	$-32 \leq h \leq 20$, $-19 \leq k \leq 19$, $-42 \leq l \leq 41$
Reflections collected	22319
Independent reflections	11226 [R_{int} = 0.0317, R_{sigma} = 0.0485]
Data/restraints/parameters	11226/0/579
Goodness-of-fit on F^2	1.010
Final R indexes [I>=2σ (I)]	R_1 = 0.0343, wR_2 = 0.0657
Final R indexes [all data]	R_1 = 0.0522, wR_2 = 0.0730
Largest diff. peak/hole / e Å^{-3}	1.73/-1.26

Interatomic distances

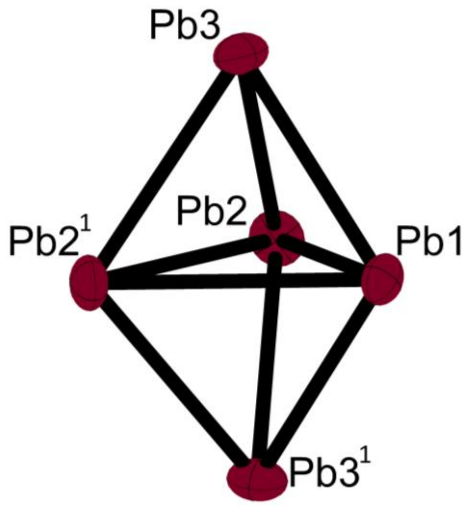

Figure S9: Anionic unit $[Pb_5]^{2-}$ in $[Rb@[2.2.2]crypt]_2[Pb_5][PPh_3]_2 \cdot NH_3$, with the corresponding distances in Table S14. Symmetry code #1: −X,+Y,1/2−Z.

Table S14: Selected interatomic distances in $[Rb@[2.2.2]crypt]_2[Pb_5][PPh_3]_2 \cdot NH_3$.

Atom	Atom	Length/Å	Atom	Atom	Length/Å	Atom	Atom	Length/Å
Pb1	Pb2[1]	3.2327(2)	Pb2	Pb3[1]	3.0025(3)	Rb1	O1	2.844(3)
Pb1	Pb2	3.2328(2)	Pb2	Pb3	2.9989(3)	Rb1	O3	2.904(3)
Pb1	Pb3	3.0037(3)	Rb1	O5	2.856(3)	Rb1	O4	2.865(3)
Pb1	Pb3[1]	3.0037(3)	Rb1	O2	2.909(3)	Rb1	N2	3.007(4)
Pb2	Pb2[1]	3.2394(3)	Rb1	O6	2.891(3)	Rb1	N1	3.006(4)

[1]1-X,+Y,1/2-Z

Structural comparison of $CaIn_2$ with $[Rb@[2.2.2]crypt]_2[Sn_5][PPh_3]_2 \cdot NH_3$ and $[Rb@[2.2.2]crypt]_2[Pb_5][PPh_3]_2 \cdot NH_3$

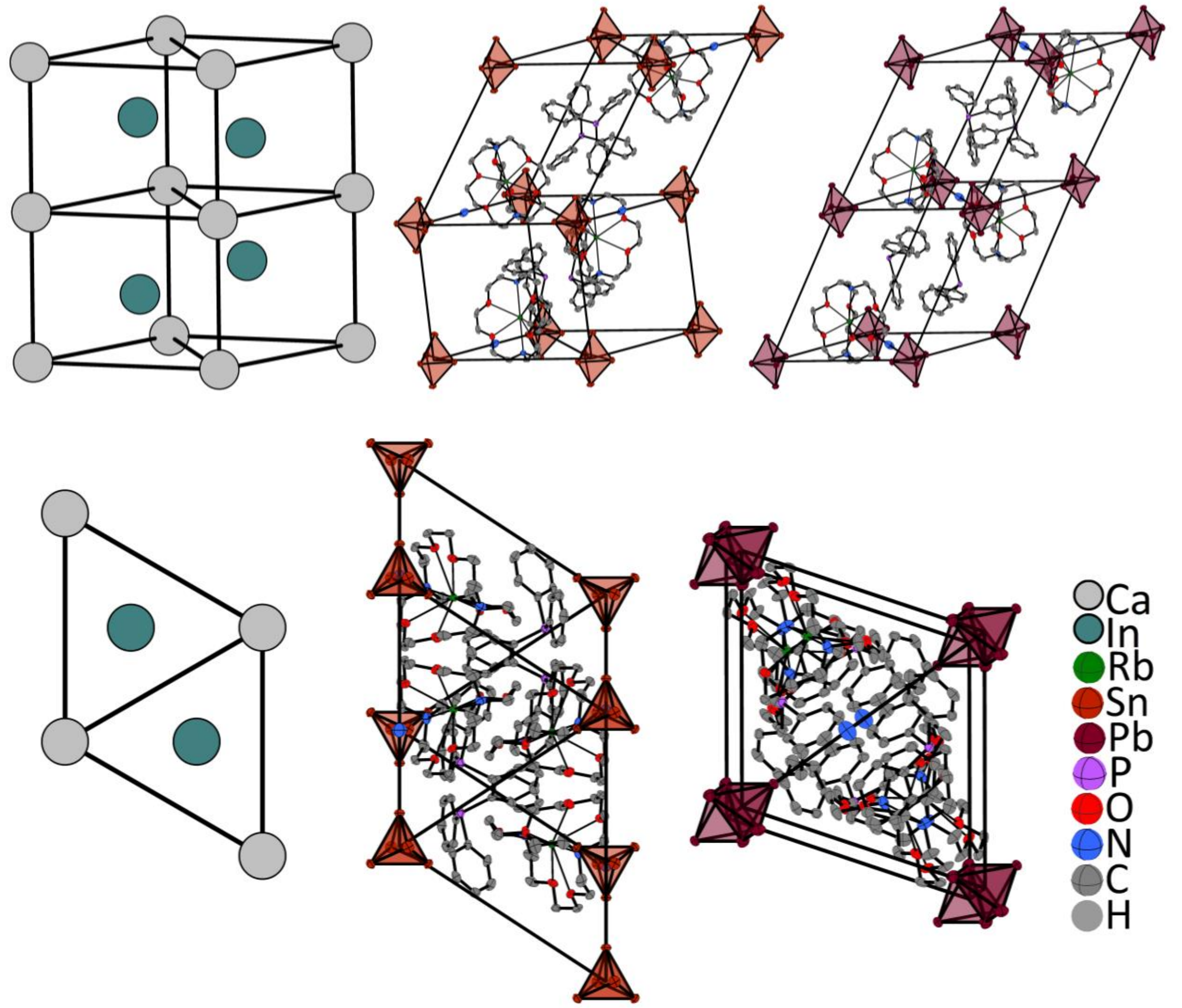

Figure S10: Structural comparison between $CaIn_2$, $[Rb@[2.2.2]crypt]_2[Sn_5][PPh_3]_2 \cdot NH_3$ and $[Rb@[2.2.2]crypt]_2[Pb_5][PPh_3]_2 \cdot NH_3$, visualizing the distorted hexagonal packing in $[Rb@[2.2.2]crypt]_2[Sn_5][PPh_3]_2 \cdot NH_3$ and $[Rb@[2.2.2]crypt]_2[Pb_5][PPh_3]_2 \cdot NH_3$. The shown section of the crystal structure is not comparable to the unit cell.

$[K@[2.2.2]crypt]_3[HSi_9][PPh_3] \cdot 5NH_3$ (8):

Synthesis:

A solid state material with the nominal composition $K_6Rb_6Si_{17}$ (30 mg, 0.025 mmol), [2.2.2]crypt (28 mg, 0.073 mmol), [18]crown-6 (9 mg, 0.037 mmol) and $Pt(PPh_3)_4$ (30 mg, 0.025 mmol) were dissolved in anhydrous liquid ammonia, yielding a orange-brown solution. After storage at 233 K for one year, yellow blocks of (8) could be obtained.

Crystallographic information:

The crystal structure was recorded on an Agilent SuperNova with microfocus Mo-X-ray source and an Eos CCD detector. The corresponding crystallographic data is listed in Table S15.

Table S15: Crystallographic data of [K@[2.2.2]crypt]$_3$[HSi$_9$][PPh$_3$] · 5NH$_3$.

Chemical formula	[K@[2.2.2]crypt]$_3$[HSi$_9$][PPh$_3$] · 5NH$_3$
CSD number	2330988
Formula weight	1848.01
Temperature/K	123(2)
Crystal system	triclinic
Space group	*P*-1
a/Å	14.1417(8)
b/Å	14.9160(9)
c/Å	26.7081(15)
α/°	105.980(5)
β/°	90.196(5)
γ/°	116.145(6)
Volume/Å^3	4810.8(5)
Z	2
ρ_{calc}g/cm^3	1.276
μ/mm^{-1}	0.335
F(000)	1980.0
Crystal size/mm^3	0.208 × 0.15 × 0.085
Radiation	Mo Kα (λ = 0.71073)
2Θ range/°	5.972 to 57.004
Index ranges	$-17 \leq h \leq 18$, $-19 \leq k \leq 20$, $-35 \leq l \leq 35$
Reflections collected	53487
Independent reflections	20719 [R_{int} = 0.0980, R_{sigma} = 0.1732]
Data/restraints/parameters	20719/12/1050
Goodness-of-fit on F^2	1.026
Final R indexes [I>=2σ (I)]	R_1 = 0.0769, wR_2 = 0.1453
Final R indexes [all data]	R_1 = 0.1639, wR_2 = 0.1845
Largest diff. peak/hole / e Å^{-3}	0.95/-0.80

Interatomic distances

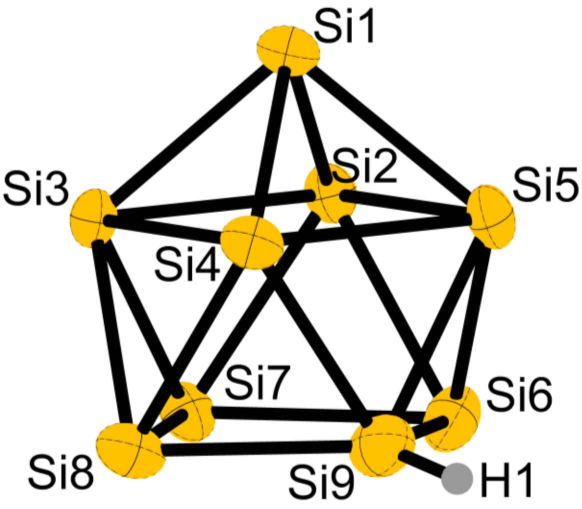

Figure S11: Anionic unit $[HSi_9]^{3-}$ in $[K@[2.2.2]crypt]_3[HSi_9][PPh_3] \cdot 5NH_3$, with the corresponding distances in Table S16.

Table S16: Selected interatomic distances in $[K@[2.2.2]crypt]_3[HSi_9][PPh_3] \cdot 5NH_3$.

Atom	Atom	Length/Å	Atom	Atom	Length/Å	Atom	Atom	Length/Å
K2	O12	2.814(3)	K3	N5	3.045(4)	Si2	Si1	2.434(2)
K2	O11	2.843(3)	K1	O1	2.772(3)	Si2	Si6	2.429(2)
K2	O7	2.858(3)	K1	O2	2.840(3)	Si5	Si4	2.708(2)
K2	O8	2.876(3)	K1	O4	2.806(3)	Si5	Si1	2.444(2)
K2	O10	2.891(3)	K1	O3	2.815(3)	Si5	Si6	2.524(2)
K2	O9	2.885(3)	K1	O5	2.763(3)	Si5	Si9	2.439(2)
K2	N3	2.971(4)	K1	N2	3.020(4)	Si7	Si8	2.5412(19)
K2	N4	2.964(4)	K1	N1	3.015(4)	Si7	Si6	2.500(2)
K3	O15	2.829(3)	Si3	Si2	2.6734(18)	Si4	Si1	2.4447(19)
K3	O14	2.817(3)	Si3	Si7	2.446(2)	Si4	Si8	2.495(2)
K3	O16	2.820(3)	Si3	Si4	2.6168(19)	Si4	Si9	2.429(2)
K3	O17	2.786(3)	Si3	Si1	2.447(2)	Si8	Si9	2.348(2)
K3	O18	2.850(3)	Si3	Si8	2.433(2)	Si6	Si9	2.351(2)
K3	O13	2.866(3)	Si2	Si5	2.604(2)	Si9	H1	1.21(6)
K3	N6	3.041(4)	Si2	Si7	2.438(2)			

References

(1) Neese, F. The ORCA Program System. *Wiley Interdiscip. Rev. Comput. Mol. Sci.* **2012**, *2* (1), 73–78. https://doi.org/10.1002/wcms.81.

(2) Neese, F. Software Update: The ORCA Program System—Version 5.0. *Wiley Interdiscip. Rev. Comput. Mol. Sci.* **2022**, *12* (5), e1606. https://doi.org/10.1002/wcms.1606.

(3) Becke, A. D. Density-functional Thermochemistry. III. The Role of Exact Exchange. *Chem. Phys.* **1993**, *98* (7), 5648–5652. https://doi.org/10.1063/1.464913.

(4) Lee, C.; Yang, W.; Parr, R. G. Development of the Colle-Salvetti Correlation-Energy Formula into a Functional of the Electron Density. *Phys. Rev. B* **1988**, *37* (2), 785–789. https://doi.org/10.1103/PhysRevB.37.785.

(5) Vosko, S. H.; Wilk, L.; Nusair, M. Accurate Spin-Dependent Electron Liquid Correlation Energies for Local Spin Density Calculations: A Critical Analysis. *Can. J. Phys.* **1980**, *58* (8), 1200–1211. https://doi.org/10.1139/p80-159.

(6) Stephens, P. J.; Devlin, F. J.; Chabalowski, C. F.; Frisch, M. J. Ab Initio Calculation of Vibrational Absorption and Circular Dichroism Spectra Using Density Functional Force Fields. *J. Phys. Chem.* **1994**, *98* (45), 11623–11627. https://doi.org/10.1021/j100096a001.

(7) Weigend, F.; Ahlrichs, R. Balanced Basis Sets of Split Valence, Triple Zeta Valence and Quadruple Zeta Valence Quality for H to Rn: Design and Assessment of Accuracy. *Phys. Chem. Chem. Phys.* **2005**, *7* (18), 3297–3305. https://doi.org/10.1039/B508541A.

(8) Barone, V.; Cossi, M. Quantum Calculation of Molecular Energies and Energy Gradients in Solution by a Conductor Solvent Model. *J. Phys. Chem. A* **1998**, *102* (11), 1995–2001. https://doi.org/10.1021/jp9716997.
